# Nutritional Characterization of Selected South African Indigenous Seed Flours and Their Impact on Bread Baking Performance

**DOI:** 10.1111/1750-3841.71382

**Published:** 2026-08-22

**Authors:** Karen de Jager, Wilma Augustyn, Thierry Regnier, Belinda Meiring

**Affiliations:** ^1^ Agricultural Research Council‐Tropical and Subtropical Crops Agro‐Processing Nelspruit South Africa; ^2^ Department of Biotechnology and Food Technology Tshwane University of Technology Pretoria South Africa; ^3^ Department of Chemistry Tshwane University of Technology Pretoria South Africa

**Keywords:** underutilized crops, nutritionally enhanced bread, composite flours, dough rheology, baking performance

## Abstract

Diets based predominantly on refined cereal staples are associated with inadequate intake of high‐quality protein and essential micronutrients. This study evaluated five underutilized South African indigenous seed flours, Lowveld chestnut (*Sterculia murex*), marula (*Sclerocarya birrea*), forest mahogany (*Trichilia dregeana*), Natal mahogany (*Trichilia emetica*), and stamvrug (*Englerophytum magalismontanum*), as functional ingredients in composite bread at 10%–20% substitution levels. Analyses included proximate composition, amino acid and mineral profiles, phenolic content, microbial quality, dough rheology (Mixolab), and bread baking performance using C‐Cell and texture analysis. The flours exhibited distinct compositional profiles, with marula characterized by high protein and lipid content and Lowveld chestnut contributing favorable essential amino acid composition. Incorporation into wheat dough reduced dough strength, especially in lipid‐rich formulations, while stamvrug increased dough stiffness. These rheological differences were also reflected in crumb structure and firmness development during storage. Marula and mahogany breads exhibited more open crumb structures and reduced firming, whereas stamvrug produced dense crumb structures and accelerated firming. Baking reduced microbial loads; however, changes during storage emphasized the need for appropriate control of raw materials and postbaking handling. Among the seed flours evaluated, marula and Lowveld chestnut provided the most favorable balance between nutritional improvement and baking performance, demonstrating the potential of underutilized indigenous seed flours as high‐value functional ingredients.

**Practical Applications:**

This research demonstrates the potential of locally available indigenous seeds for developing nutritionally enhanced bread to benefit communities and the food industry.

## 1 Introduction

Despite global advances in food production, hunger and malnutrition remain persistent challenges. In 2024, approximately 638 to 720 million (7.8% to 8.8%) people globally experienced hunger (FAO et al. 2025). According to a report by (FAO et al. 2022), 20.2% of Africa's population faced hunger in 2021. During 2024, 2.60 billion (31.9%) worldwide were unable to afford a healthy diet that sustains energy and most nutrient requirements (FAO et al. 2025). Beyond caloric intake, widespread micronutrient deficiencies, often termed “hidden hunger,” continue to compromise public health, leading to impaired physical and cognitive development, reduced immunity, and increased susceptibility to disease, particularly among vulnerable populations such as young children and women of reproductive age (Stevens et al. 2022).

High rates of undernutrition and micronutrient deficiencies coexist with rising rates of overweight and obesity, particularly among vulnerable populations in rural areas and informal settlements (Labadarios et al. 2005). A significant contributing factor to this nutritional inadequacy, especially for young children, is the heavy reliance on inexpensive, refined staple foods such as maize meal and commercially produced bread (Oldewage‐Theron and Kruger 2008). While providing essential calories, these staples often lack the necessary diversity of micronutrients (vitamins and minerals) and macronutrients (e.g., quality protein, healthy fats) crucial for optimal growth and development, even with mandatory fortification programmes (Steyn et al. 2008; Galani et al. 2022). This limited dietary diversity, particularly for infants and young children during critical growth windows, can lead to irreversible developmental consequences (Oldewage‐Theron and Kruger 2008). Semba et al. (2016) reported that children at high risk of stunting exhibit markedly lower circulating concentrations of all essential amino acids, suggesting that their dietary intake of essential amino acids may be insufficient to support optimal growth. To address these challenges, researchers have investigated incorporating nutrient‐dense ingredients, such as seeds, nuts, and tubers, into food formulations. Breads enriched with sunflower seeds, pumpkin seeds, and legumes have demonstrated improved protein quality and mineral content (Czajkowska–González et al. 2021). For instance, cold‐pressed flaxseed oil cake, rich in protein (38.0%–47.3%), omega‐3 fatty acids (58.5%–59.7%), dietary fiber, and minerals (e.g., calcium 3.3–3.8 mg/g, phosphorus 6.4–8.2 mg/g) can be included in bread formulations at 10%–15% levels for nutritional enhancement (Ogunronbi et al. 2011). Addressing these nutritional gaps requires innovative approaches to enhance the nutrient density of staple foods, especially those widely consumed and culturally acceptable (Amagloh et al. 2012). Bread, as a staple food, offers an ideal vehicle for fortification due to its ubiquity, affordability, and compatibility with composite flours, enabling integration of nutrient‐rich additives without significant alterations to taste or production processes (Yusufali et al. 2012).

While other regions have successfully developed and commercialized nutrient‐enhanced breads using various alternative ingredients, South Africa has somewhat lagged in fully exploring the potential of its diverse indigenous plant resources for this purpose (Amoah et al. 2020). South Africa possesses a wealth of underutilized indigenous fruits and seeds, many of which are known for their exceptional nutritional profiles, including high levels of essential fatty acids, proteins, dietary fiber, vitamins, and minerals (Mabhaudhi et al. 2016; Mabhaudhi et al. 2017; Van Wyk and Gericke 2018). Integrating underutilized crops into agriculture can enhance biodiversity, reduce dependence on monocultures, and support the development of a more nutritious, resilient, and sustainable global food system by valuing and harnessing nature's diversity (Purba and Krishnaswamy 2025). Within this context, indigenous seed resources that are already traditionally used as foods represent promising candidates for incorporation into widely consumed staple products such as bread.

Marula kernels are enclosed within the hard, woody stones of the marula fruit and are traditionally consumed by local communities either raw or cooked with maize meal (Boon and Pooley 2010). The kernels are also valued as a source of edible oil used for culinary and cosmetic applications (Van Wyk and Gericke 2018). The seeds of forest mahogany and Natal mahogany are consumed after removal of the seed coat, often as a milky extract prepared by soaking the seeds in water and eaten with leafy vegetables, while the extracted oils are used in soap manufacture and cosmetic products (Boon and Pooley 2010). The oil‐rich seeds of Lowveld chestnut are commonly consumed fresh or roasted and have been reported to contain approximately 14% protein, together with appreciable phenolic content and antioxidant activity (Joffe and Oberholzer 2012; Regnier et al. 2017). Despite their traditional use and favorable nutritional characteristics, these indigenous seed resources remain largely underutilized in formal food product development. Their incorporation into wheat‐based products may enhance nutritional quality by increasing protein, amino acid, mineral, and bioactive compound content, but may also affect dough rheology and bread quality due to differences in protein, starch, fiber, and lipid composition. Consequently, understanding the balance between nutritional enhancement and technological performance is essential for their successful utilization in bakery products.

**FIGURE 1:**
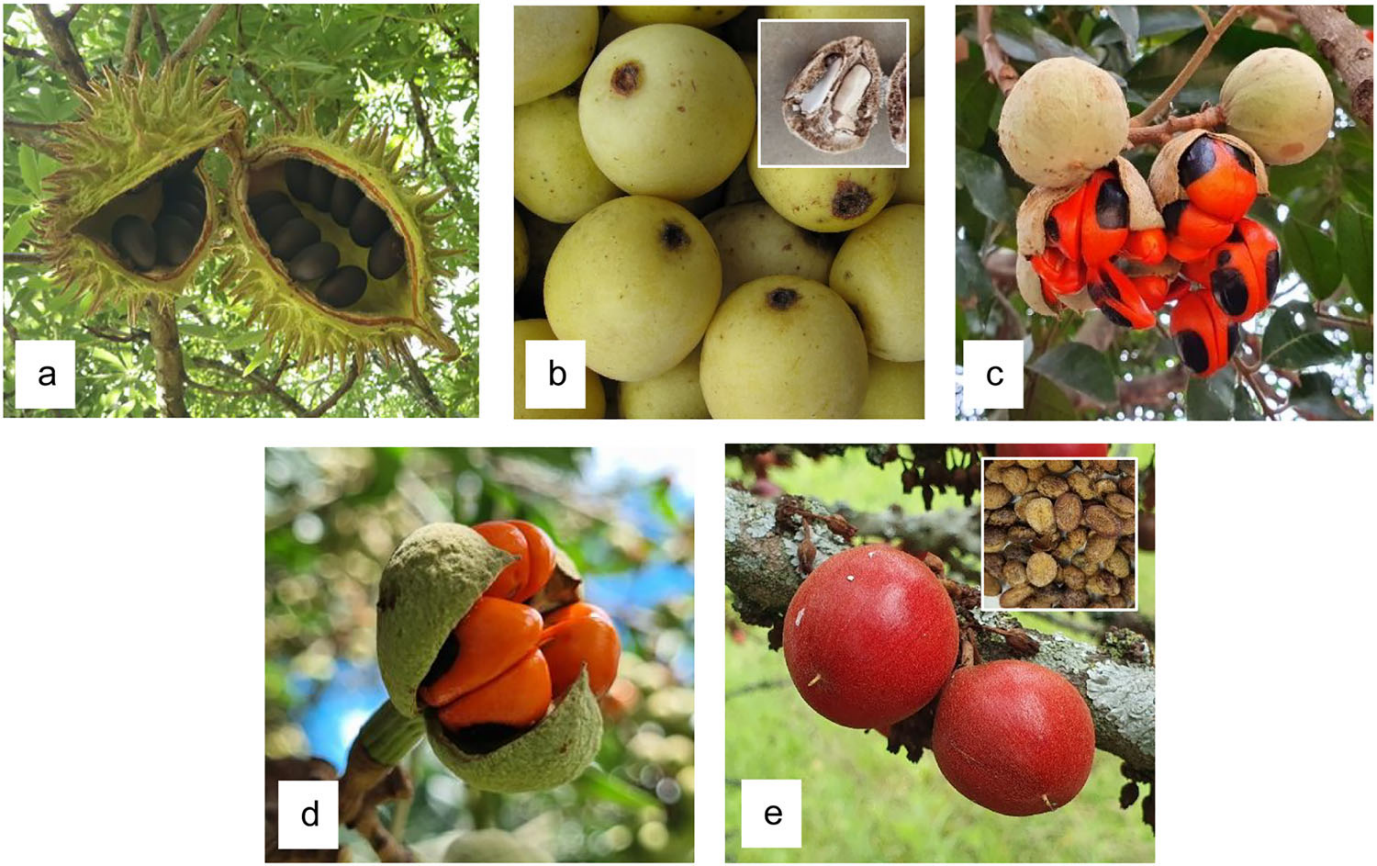
Selected indigenous seed sources used in this study: Lowveld chestnut (a), marula (seeds insert) (b), forest mahogany (c), Natal mahogany (d), and stamvrug (seeds insert) (e).

In this study, *Sterculia murex* (Lowveld chestnut), *Sclerocarya birrea* (marula), *Trichilia dregeana* (forest mahogany), *Trichilia emetica* (Natal mahogany), and *Englerophytum magalismontanum* (stamvrug) (Figure 1) were selected based on the high nutrient density of their seeds, their local abundance within South African ecosystems, and their current underutilization despite offering notable sustainability advantages. It was hypothesized that partial substitution of wheat flour with these indigenous seed flours would improve the nutritional profile of bread while producing measurable changes in dough rheology and baking performance. Therefore, the objectives of this study were to (i) evaluate the nutritional composition, phenolic content and microbiological quality of the selected indigenous seed flours; and (ii) determine their effects on dough rheology and bread quality characteristics, including baking loss, color, crumb structure, and firmness during storage.

## 2 Materials and Methods

### 2.1 Chemicals, Reagents, Microbiological Media and Bread Ingredients

All chemicals and reagents of analytical grade used in the study were purchased from internationally recognized companies [Radchem Pty LTD, South Africa, Merck (Sigma‐Aldrich, Millipore, Sigma), Thermo Fisher Scientific (Oxoid), BD Biosciences, Bio‐Rad Laboratories]. For the bread formulation, all ingredients were sourced locally from reputable suppliers. The commercial white bread wheat flour was purchased from RCL Foods Milling (Ruto Mills, Pretoria, South Africa), and the yeast was obtained from Anchor Yeast (Johannesburg, South Africa), while the sunflower oil, sugar and salt were purchased from Pick n Pay (Pretoria, South Africa). The Format (SB 5% Bread Premix) that was used as a commercial bread additive was obtained from Aries (PTY) LTD (Johannesburg, South Africa).

### 2.2 Seed Sample Collection and Preparation

Seeds were harvested in 2021 from the miscellaneous orchard of the Agricultural Research Council—Tropical and Subtropical Crops (ARC‐TSC) in Mpumalanga, South Africa. Mature green marula fruits were collected from the ground, whereas all other selected fruits were harvested directly from the tree at the ripe stage, as determined by their color. After harvesting, the seeds were spread out on trays and air‐dried for two weeks. Seeds were subsequently further dried at 40 ± 1°C in an air oven (Labcon 5016LC, Tshwane, South Africa) and then ground using a Hamilton Beach Commercial blender (model HBF500S, Hamilton Beach Brands Inc., VA, USA). Seeds with a high oil content, such as those of forest mahogany and marula, were further crushed using a porcelain pestle and mortar following blending to homogenize the flour. The resulting flour was sieved through an aperture size of 250 µm. Composite flours were prepared by separately blending white bread wheat flour with each indigenous seed flour at 10%, 15%, and 20% (w/w) substitution levels. Each formulation consisted of wheat flour and a single indigenous seed flour; no mixed‐seed flour formulations were prepared. These formulations were subsequently subjected to standardized baking trials.

### 2.3 Microbiological Characterization of Seeds and Bread

The microbiological safety and stability of flours and bread samples were assessed through quantitative enumeration and molecular identification. Total viable counts (TVC) were determined according to the AOAC 990.12 protocol, while yeast and mold counts followed ISO 21527‐1 (2008).

#### 2.3.1 Sample Preparation and Enumeration

Samples were prepared by homogenizing 10 g of flour or 1 g of bread (day 1 and day 5 of shelf life) with sterile Ringer's solution in a stomacher bag (260 rpm, 3 min). Serial dilutions were prepared in Ringer's solution, ranging from 10^−1^ to 10^−12^ for flours and 10^−1^ to 10^−5^ for bread samples. Enumeration was performed using the pour plate technique. One ml of each dilution was transferred to sterile Petri dishes with Plate Count Agar (PCA, Biolab, South Africa) for bacterial counts or Malt Extract Agar (MEA) for yeast and mold counts. Bacterial plates (PCA) were incubated at 37°C for 48 h, while MEA plates were monitored for yeast and mold growth (25°C for 5 days). Microbial load was expressed as colony‐forming units per gram (CFU/g).

#### 2.3.2 Isolation and Identification

Pure cultures were established by transferring specific colonies onto Potato Dextrose Agar (PDA) for yeast and molds and Nutrient Agar (NA) for bacteria. Cultures were incubated until distinct colonies developed, with purity verified through macroscopic and microscopic examination. Molecular identification was conducted at Inqaba Biotec (Pretoria, South Africa). Genomic DNA was extracted from the isolates, followed by PCR amplification of the Internal Transcribed Spacer (ITS) region using conserved primers. The resulting amplicons were sequenced using Next Generation Sequencing (NGS) and identified by comparing sequences against curated databases, including GenBank and UNITE.

### 2.4 Nutritional Composition

#### 2.4.1 Moisture Content

The moisture contents of Lowveld chestnut, marula, forest mahogany, Natal mahogany and stamvrug flours were determined by oven‐drying at 105°C for 16 h. The percentage weight loss was reported as the moisture content (AOAC Official Methods 934.01 and 930.15). The moisture content of the composite flours was determined using a Kern MLB N electronic moisture meter (Balinger, Germany). The samples (1 g) were spread evenly on an aluminum sample dish, and the analysis was carried out at 120°C until constant weight (≤1 mg change in weight within 60 s).

#### 2.4.2 Protein Content

The protein content of the samples was determined using the Kjeldahl method, which quantifies total organic nitrogen. The organic material was digested with hot, concentrated sulfuric acid, to which a catalyst mixture was added to elevate the boiling point. This process converted all nitrogen to ammonia, which was subsequently measured via titration (AOAC Official Method 954.01). A factor of 6.25 was used to convert the nitrogen content to percentage protein.

#### 2.4.3 Lipid Extraction

Lipid extraction was performed using a VELP SER 148 semiautomatic solvent extractor (VELP Scientifica, Usmate Velate, Italy) with petroleum ether (40–60°C) as the extraction solvent (Anderson 2019). Approximately 10 g of finely ground sample was placed in predried cellulose thimbles, and 70 mL petroleum ether was used per extraction. Extraction was carried out using a programmed sequence consisting of immersion (30 min at 130°C), washing under reflux (50 min at 130°C), and solvent recovery (30 min at 130°C). Following extraction, residual solvent was removed by drying at 70°C to constant weight. Lipid content was determined gravimetrically (Anderson 2019) and calculated using the following equation:

$$\mathrm{Lipid}\;\mathrm{Yield}\;\;\left(\%\right)=\frac{\;\mathrm{Weight}\;\mathrm{of}\;\mathrm{extracted}\;\mathrm{lipid}\;\left(g\right)}{\mathrm{Weight}\;\mathrm{of}\;\mathrm{seed}\;\mathrm{flour}\;\mathrm{sample}\;\left(g\right)}\;\times \;100$$

#### 2.4.4 Ash Content

The ash content of the samples was determined by subjecting them to a temperature of 550°C overnight to completely combust all organic matter. The remaining inorganic residue, known as ash, was then calculated as a percentage of the original sample mass (AOAC Official Method 942.05).

#### 2.4.5 Amino Acid Content

Amino acids were quantified following acid hydrolysis of flour proteins with 6 M HCl at 110°C for 24 h under nitrogen. Sulphur amino acids were determined after performic acid oxidation before hydrolysis, while tryptophan was measured separately using alkaline hydrolysis due to its acid lability. Norleucine was included as an internal standard.

Hydrolysates were subjected to precolumn derivatization using the Aminotag system, which provides high sensitivity and automated handling of amino acid samples (Cunico et al. 1986). Derivatized amino acids were separated on a reversed‐phase C18 column with gradient elution and detected by UV and fluorescence. Quantification was achieved using calibration curves prepared from amino acid standards processed identically. For confirmation, amino acids were also derivatized with 9‐fluorenylmethyl chloroformate (FMOC‐Cl) and analyzed by reversed‐phase HPLC (Shimadzu, LC‐20A, Kyoto, Japan), with detection by UV and fluorescence (Einarsson et al. 1983). Concentrations were calculated relative to the internal standard and expressed as g/100 g protein.

Amino Acid Score (AAS) was calculated using the age‐specific reference amino acid patterns of the World Health Organisation/Food and Agriculture Organisation/United Nations University (WHO et al. 2007), according to the following equation:

$$\begin{array}{ccc} & & \mathrm{AAS}\;=\\ & & \frac{\mathrm{mg}\;\mathrm{of}\;\mathrm{essential}\;\mathrm{amino}\;\mathrm{acid}\;\mathrm{per}\;g\;\mathrm{test}\;\mathrm{protein}}{\mathrm{mg}\;\mathrm{of}\;\mathrm{the}\;\mathrm{same}\;\mathrm{essential}\;\mathrm{amino}\;\mathrm{acid}\;\mathrm{per}\;g\;\mathrm{protein}\;\mathrm{in}\;\mathrm{the}\;\mathrm{reference}\;\mathrm{pattern}} \end{array}$$

#### 2.4.6 Mineral Composition

Mineral analysis was performed according to the standard procedures of AgriLASA (2007) and AOAC (2005). Samples were oven‐dried (60°C, 24 h) and milled to pass through a 1 mm sieve. For the determination of Ca, Mg, K, Na, Zn, Cu, Mn, and Fe, 0.5 g of sample underwent wet acid digestion using a HNO_3_/HClO_4_ mixture (4:2 v/v) at 100°C for 2 h, followed by heating at 180°C until clear. Resulting digests were diluted to 25 mL with de‐ionized water and quantified using a Flame Atomic Absorption Spectrometer (AA200, Varian, Australia).

Nitrogen, phosphorus, and boron were determined using an Auto Analyzer 3 (Bran and Luebbe, Germany) following sulfuric acid and hydrogen peroxide digestion (300°C). Colorimetric measurements were performed at 660 nm for N and P (using sodium salicylate and ammonium molybdate reagents, respectively) and 420 nm for B (using azomethine‐H). Element concentrations were calculated against standard curves and expressed as g/kg (N, P, K, Ca, Mg) or mg/kg (Zn, Cu, Mn, Fe, B), consistent with established protocols for plant tissue analysis (Miller 1998).

### 2.5 Phenolic Content

Flours were defatted using three cycles of solvent washing with hexane, with the exception of marula seeds, for which petroleum ether was used (Régnier and Macheix 1996; De Ascensao and Dubery 2003). The defatted material was dried in an air oven (Labcon 5016LC, Tshwane, South Africa) at 40°C for 48 h and stored in a desiccator at ambient temperature until extraction. Phenolic compounds were extracted using solvents of varying polarity, including water, aqueous methanol, aqueous acetone, and a methanol:acetone:water mixture, as described in previous studies (Makkar et al. 1993). Extractions were performed in triplicate, and supernatants were combined and stored at 4°C until analysis. Total phenolic content was quantified using the Folin–Ciocalteu (Bray and Thorpe 1954), as modified by Du Plooy et al. (2009), with absorbance measured at 765 nm and results expressed as gallic acid equivalents per gram of dried flour.

### 2.6 Dough Rheology

The rheological properties of the bread dough were evaluated in accordance with the standard AACC 54‐60.01 method. A Chopin Mixolab instrument utilizing the Chopin+ protocol (Chopin, Villeneuve‐la‐Garenne, France) was employed for the analysis. The hydration level was maintained at 60% while the flour moisture basis was consistently set at 14%.

### 2.7 Baking Performance

#### 2.7.1 Baking Methods

The bread dough was prepared using 500 g of flour (white bread wheat flour or composite flours prepared as described in 2.2), 5 g of sugar, 5 g of Format 5% premix (a commercial bread baking premix containing emulsifiers, enzymes and ascorbic acid), 7 g of salt, 18.8 g of instant dried yeast, 300 mL of water, and 15 mL of sunflower oil. The ingredients were initially mixed at a low speed setting for 2 min using a planetary dough mixer (Ancor Econo 20 L, Ancor, Johannesburg, South Africa), followed by a high mixing phase of 8 min. Dough portions of 195 g were kneaded manually for 3 min. The dough balls were flattened, rolled, and shaped into mini bread pans (100 mm × 55 mm), then rested for 8 min at room temperature, followed by proofing in a Milano Minuetto Nasi 4.6 proofing cabinet (Milano Forni, Milano, Italy) for 45 min at 40°C with 85% relative humidity. The proofing time was established during preliminary baking trials, where 45 min resulted in sufficient dough expansion before baking, and was subsequently applied to all formulations. The loaves were subsequently baked at 230°C for 28 min in a Minuetto Nasi 4.6 oven (Milano forni, Milano, Italy).

#### 2.7.2 Color

The color of the crust and crumb was measured using a Lovibond colorimeter (LC100, United Kingdom), based on the CIELAB color space (L*, a*, b*). The L* value represents lightness, a* denotes the red–green axis, and b* corresponds to the yellow–blue axis. The total color difference Δ*E* was calculated using the following equation (Jha 2010):

$$\Delta E={\left(\Delta L^{2}+\Delta a^{2}+\Delta b^{2}\right)}^{0.5}$$

#### 2.7.3 Baking Loss

Each loaf was weighed after cooling to room temperature. The baking loss (%) was calculated by using the following equation:

$$\mathrm{Baking}\;\mathrm{loss}\;=\frac{\mathrm{Dough}\;\mathrm{mass}\;\left(g\right)-\mathrm{Bread}\;\mathrm{mass}\;\left(g\right)}{\mathrm{Dough}\;\mathrm{mass}\;\left(g\right)}\;\;\times \;100$$

#### 2.7.4 Firmness

The bread was sliced vertically using an electric bread slicer to ensure uniform slices of 1 cm thickness. The slices were placed in sealed polyethylene bags (Ziplock, SC Johnson, Bay City, Michigan, USA) and frozen at −20°C (Ogunronbi et al. 2011). Bread samples were frozen to facilitate experimental workflow and ensure that all treatments were analyzed under standardized conditions. Before measurement, samples were thawed at room temperature and texture analysis was conducted (day 1), while additional thawed slices were stored at 25°C under sealed conditions and analyzed on day 5 to assess changes in firmness during storage.

Two slices of bread were stacked to determine the firmness of the bread, using a TA‐XTPlus texture analyzer (Stable Micro Systems, Surrey, UK) according to AACC Method 74‐09.01 (AACC, 2010) with the modification that a 20 mm Perspex cylindrical probe (model SMS P/20 P) was used to ensure full probe‐sample contact of the small slices of the mini loaves.

#### 2.7.5 C‐Cell Imaging

Bread loaves were sectioned perpendicular to the base using a food slicer (Taurus Food Slicer Steel Brushed 150 W, Cut Master, Cape Town, South Africa), yielding uniform slices of 1 cm thickness. A slice from the mid‐portion of each bread loaf was selected for evaluation. Crumb structure was assessed using a C‐Cell imaging system (Calibre Control, United Kingdom) in accordance with AACC Method 10‐18.01.

### 2.8 Statistical Analysis

All experimental analyses were performed in triplicate. The resulting datasets were subjected to analysis of variance (ANOVA), and mean separations were assessed using Fisher's protected least significant difference (LSD) test at a 5% significance threshold, implemented in Genstat version 22.1 (VSN International Ltd., Hemel Hempstead, UK).

## 3 Results and Discussion

### 3.1 Microbiological Characterization of Indigenous Seed Flours

Evaluation of the raw indigenous seed flours revealed high microbial loads, with total plate counts (TPC) predominantly in the order of 10^1^ ^3^ CFU/g, and yeast and mold counts reaching up to 10^1^ ^3^–10^14^ CFU/g (Table 1). Lowveld chestnut and Natal mahogany exhibited the highest bacterial loads at 6.3 × 10^13^ CFU/g and 5.5 × 10^13^ CFU/g, respectively, while stamvrug showed the highest yeast and mold contamination (>10^14^ CFU/g). In contrast, marula flour consistently demonstrated substantially lower microbial loads, with both TPC and yeast and mold counts in the order of 10^7^ CFU/g, representing a difference of approximately 6–7 log units relative to the most contaminated flours. The counts observed in this study are several orders of magnitude higher than those typically reported for cereal flours and low‐moisture foods. Wheat flour generally exhibits TPC values in the range of 1–6 log CFU/g, with yeast and mold counts typically below 5 log CFU/g (Rose et al. 2012; Brar and Danyluk 2018). Similarly, nuts and oilseed systems, which are more comparable in composition and water activity, are generally reported to contain relatively low microbial loads under controlled processing conditions, as their low water activity limits microbial growth, although survival of environmental microflora remains possible (Brar and Danyluk 2018). The substantially elevated counts in the present study, therefore, indicate significant pre‐ and postharvest contamination, likely arising from environmental exposure (soil, plant surfaces, air) as well as the traditional postharvest handling processes employed in this study.

**TABLE 1:** Microbial counts (total plate count and yeast and mold, CFU/g) of indigenous seed flours and composite breads at days 1 and 5 of storage.

Flour	Total plate count, CFU/g	Yeast and mold, CFU/g
Lowveld chestnut	6.3 × 10^13^	3.9 × 10^13^
Marula	6.3 × 10^7^	3.1 × 10^7^
Forest mahogany	2.5 × 10^13^	3.5 × 10^13^
Natal mahogany	5.5 × 10^13^	3.2 × 10^13^
Stamvrug	2.9 × 10^13^	>3.0 × 10^14^

Bread sample	%	Total plate count (CFU/g)		Yeast and mold (CFU/g)	
		Day 1	Day 5	Day 1	Day 5
Control	0%	Nd	9.8 × 10^7^	Nd	5.9 × 10^5^
Lowveld chestnut	10%	Nd	6.2 × 10^7^	Nd	9.4 × 10^3^
	15%	Nd	2.4 × 10^4^	Nd	2.0 × 10^3^
	20%	Nd	7.5 × 10^3^	Nd	3.4 × 10^3^
Marula	10%	Nd	4.8 × 10^4^	Nd	Nd
	15%	Nd	2.6 × 10^2^	Nd	1.6 × 10^2^
	20%	Nd	4.8 × 10^2^	Nd	1.0 × 10^2^
Forest mahogany	10%	Nd	4.3 × 10^6^	Nd	2.8 × 10^3^
	15%	Nd	2.4 × 10^4^	Nd	2.1 × 10^2^
	20%	Nd	Nd	Nd	Nd
Natal mahogany	10%	Nd	2.0 × 10^2^	Nd	1.7 × 10^2^
	15%	Nd	2.3 × 10^2^	Nd	4.3 × 10^2^
	20%	Nd	7.7 × 10^4^	Nd	3.0 × 10^2^
Stamvrug	10%	Nd	2.3 × 10^2^	Nd	2.9 × 10^2^
	15%	Nd	6.2 × 10^3^	Nd	1.8 × 10^3^
	20%	Nd	1.8 × 10^4^	Nd	1.1 × 10^3^

*Note*: Nd, not detectable using the plate count method.

The microbial profiles obtained (Table S1) were dominated by environmental‐ and storage‐associated organisms, including species of *Candida*, *Aspergillus*, *Penicillium*, and *Rhizopus*. These genera are commonly associated with plant materials, soil, and storage environments and are frequently reported in nuts and cereal systems (Magallanes López and Simsek 2021; Brar and Danyluk 2018). Interestingly, marula flour, despite having the lowest counts, exhibited greater diversity of isolates, whereas flours from Lowveld chestnut and mahogany species showed higher counts but fewer taxa, suggesting that microbial load and diversity are not necessarily directly related. The observed variation may reflect both environmental exposure and intrinsic chemical characteristics of the seeds. No foodborne pathogens such as *Salmonella*, *Escherichia coli*, or *Staphylococcus aureus* were identified among the isolates. Overall, these results provide a quantitative microbiological characterization of these indigenous seed flours, establishing a critical baseline for future studies and supporting their potential development as safe and functional food ingredients.

### 3.2 Microbial Quality of Bread

While the raw flours primarily support microbial survival due to low water activity, incorporation into dough during mixing and fermentation creates a hydrated, nutrient‐rich environment conducive to microbial proliferation (Brar and Danyluk 2018; Vermelho et al. 2024). Despite the high microbial loads observed in the raw flours, baking resulted in a substantial reduction in microbial populations across all formulations (Table 1). This is consistent with the well‐established role of baking as a lethality step, as internal crumb temperatures typically reach approximately 98–99°C (Tank et al. 2014), resulting in the inactivation of most vegetative microorganisms (Viljoen and Von Holy 1997). The low microbial counts observed on day 1 across all bread samples confirm the effectiveness of the baking process in mitigating the high initial contamination associated with the raw flours. The microorganisms detected in the bread (day 5) are most likely attributable to heat‐resistant spores, particularly from *Bacillus* spp., or to postbaking contamination during cooling, slicing, or handling.

After five days of storage, microbial counts increased across most formulations but differed markedly between the wheat control and the composite breads (Table 1). The wheat control exhibited the highest microbial load, with total plate counts reaching approximately 7–8 log CFU/g, and yeast and mold counts reaching approximately 5–6 log CFU/g. In contrast, composite breads containing indigenous seed flours generally maintained lower counts, predominantly in the range of 2–5 log CFU/g, indicating reduced microbial proliferation. Increasing substitution levels in forest mahogany breads corresponded with progressive reductions in microbial counts, suggesting a potential inhibitory effect associated with the seeds’ intrinsic composition. Plant‐derived compounds, including phenolics and other secondary metabolites, are known to exert antimicrobial activity by disrupting microbial cell membranes and interfering with metabolic pathways (De Rossi et al. 2025).

The microbial profiles of the bread samples after five days of storage revealed a diverse range of fungal and bacterial species across all formulations (Table S1). The wheat control was dominated by *Cladosporium* and *Penicillium* species, whereas the composite breads contained a broader range of microorganisms, including *Bacillus*, *Aspergillus*, and *Penicillium*. Such genera are widely recognized as typical spoilage microbiota in bread (Viljoen and Von Holy 1997; György and Laslo 2024). The presence of *Bacillus* species is of particular relevance, as these spore‐forming bacteria are commonly associated with cereal products and can survive baking due to their heat‐resistant endospores, subsequently germinating during storage to cause rope spoilage (Pacher et al. 2022). Filamentous fungi such as *Aspergillus*, *Penicillium*, and *Cladosporium* are well established as primary agents of mold spoilage in bread, typically arising from postbaking environmental contamination rather than survival through baking (Viljoen and Von Holy 1997; György and Laslo 2024).

Several of the genera identified also include species with recognized toxigenic potential. For example, *Penicillium citrinum* is known to produce citrinin, a nephrotoxic compound (Ollinger et al. 2024), while species within *Aspergillus*, such as *A. nomiae*, have been associated with aflatoxin production under favorable growth conditions (Reis et al. 2022). In addition, *Alternaria* species are capable of producing toxins such as tenuazonic acid, which has been linked to adverse health effects (Kumari and Tirkey 2019). Although the detection of these taxa does not confirm toxin production, their presence highlights the potential for mycotoxin formation under favorable conditions and reinforces the importance of stringent quality control during processing and storage. The overall findings demonstrate that microbial outcomes in composite breads reflect the combined influence of raw material quality, processing conditions, and postbaking handling, underscoring the need for integrated control of microbial dynamics to ensure product safety and shelf life.

### 3.3 Nutritional Composition

#### 3.3.1 Moisture

Moisture levels were low in marula (4.07%), Natal mahogany (4.34%), and forest mahogany (4.45%) flours (Table 2). These lower moisture contents are advantageous for shelf life and storage stability. In contrast, the higher moisture levels in Lowveld chestnut (9.37%) and stamvrug (8.62%) flours may necessitate additional drying and specific storage conditions to prevent microbial spoilage.

**TABLE 2:** Nutritional composition and amino acid profiles of Lowveld chestnut, marula, forest mahogany, Natal mahogany, and stamvrug seeds (*n* = 3).

	Lowveld chestnut	Marula	Forest mahogany	Natal mahogany	Stamvrug
Moisture (%)	9.37 ^a^ ±0.06	4.07 ^e^ ±0.06	4.45 ^c^ ±0.05	4.34 ^d^ ±0.03	8.62 ^b^ ±0.03
Lipids (%)	7.5 ^c^ ±0.48	34.5 ^b^ ±0.86	41.8 ^a^ ±3.02	40.8 ^a^ ±4.01	4.9 ^d^ ±0.59
Protein (%)	13.2 ^c^ ±0.10	26.8 ^a^ ±0.71	8.6 ^d^ ±0.23	13.9 ^b^ ±0.10	5.5 ^e^ ±0.10
Nonessential amino acids (g/100 g protein)					
Arginine (Arg)	19.2 ^a^ ±0.46	17.6 ^b^ ±0.09	9.0 ^d^ ±0.24	11.7 ^c^ ±0.14	11.4 ^c^ ±0.21
Serine (Ser)	4.6 ^c^ ±0.17	4.7 ^c^ ±0.02	5.9 ^b^ ±0.20	6.4 ^a^ ±0.08	5.9 ^b^ ±0.32
Aspartic acid (Asp)	8.8 ^c^ ±0.29	8.1 ^c^ ±0.04	13.4 ^a^ ±0.42	12.6 ^ab^ ±0.17	11.4 ^b^ ±0.21
Glutamic acid (Glu)	16.9 ^c^ ±0.33	25.2 ^a^ ±0.14	12.7 ^d^ ±0.38	11.5 ^e^ ±0.15	18.0 ^b^ ±0.48
Glycine (Gly)	3.6 ^c^ ±0.13	5.2 ^b^ ±0.02	5.6 ^a^ ±0.12	5.6 ^a^ ±0.04	5.7 ^a^ ±0.11
Alanine (Ala)	4.0 ^b^ ±0.04	3.0 ^e^ ±0.02	3.8 ^c^ ±0.13	3.6 ^d^ ±0.08	4.4 ^a^ ±0.11
Tyrosine (Tyr)	1.3 ^b^ ±0.19	1.5 ^b^ ±0.00	2.0 ^a^ ±0.07	1.9 ^a^ ±0.04	1.6 ^b^ ±0.28
Proline (Pro)	2.7 ^e^ ±0.00	3.1 ^d^ ±0.02	12.4 ^a^ ±0.07	7.9 ^b^ ±0.00	4.4 ^c^ ±0.11
Hydroxy proline (Hyp)	0.2 ^d^ ±0.00	0.1 ^d^ ±0.02	1.1 ^b^ ±0.07	0.8 ^c^ ±0.00	1.7 ^a^ ±0.11
Cysteine (Cys)	0.8 ^d^ ±0.01	6.3 ^a^ ±0.09	3.1 ^c^ ±0.01	4.0 ^b^ ±0.21	3.8 ^b^ ±0.06
Essential amino acids (g/100 g protein)					
Threonine (Thr)	3.7 ^b^ ±0.13	2.4 ^d^ ±0.02	3.5 ^c^ ±0.12	3.5 ^c^ ±0.04	5.7 ^a^ ±0.11
Methionine (Met)	1.8 ^a^ ±0.08	1.5 ^ab^ ±0.04	0.8 ^d^ ±0.38	1.0^ cd^ ±0.11	1.3 ^bc^ ±0.32
Valine (Val)	4.5 ^e^ ±0.04	5.2 ^d^ ±0.04	6.7 ^b^ ±0.18	7.4 ^a^ ±0.08	6.0 ^c^ ±0.11
Phenylalanine (Phe)	2.8 ^d^ ±0.08	4.0 ^a^ ±0.02	3.8 ^b^ ±0.07	3.9 ^ab^ ±0.04	3.5 ^c^ ±0.11
Leucine (Leu)	4.4 ^d^ ±0.12	5.4 ^c^ ±0.06	6.0 ^b^ ±0.18	6.2 ^ab^ ±0.08	6.3 ^a^ ±0.11
Isoleucine (Ile)	3.1 ^d^ ±0.09	4.2 ^b^ ±0.21	5.7 ^a^ ±0.18	5.6 ^a^ ±0.07	3.9 ^c^ ±0.11
Histidine (His)	6.9 ^a^ ±0.43	2.7 ^d^ ±0.16	2.9 ^d^ ±0.42	5.4 ^b^ ±0.00	3.5 ^c^ ±0.28
Lysine (Lys)	7.0 ^a^ ±0.50	2.3 ^c^ ±0.02	4.0 ^b^ ±0.07	4.1 ^b^ ±0.07	3.8 ^b^ ±0.18
Nonessential amino acid (NEAA): Essential amino acid (EAA) ratio					
	1.8	2.7	2.0	2.1	1.8

*Note*: Different letters in the same row indicate significant differences between samples by Fisher's protected least significant difference (LSD) test (*p* < 0.05) followed by ±SD.

#### 3.3.2 Protein and Amino Acids

The protein content of the indigenous seed flours (Table 2) varied significantly (*p* < 0.05) among the five species evaluated. Marula flour had the highest protein content (26.8%), although slightly lower than the 36.4% (Glew et al. 2004) and 28.4% (Attiogbe and Abdul‐Razak 2016) reported in the literature. The high protein content indicates the potential of marula flour as a valuable plant‐based protein source for enriching cereal‐based products. Lowveld chestnut contained 13.2% protein, which is consistent with the value of 14.5% reported by Regnier et al. (2017). Similarly, Natal mahogany exhibited a protein content of 13.9%, which falls within the broad range (5.2–25.6%) reported in the literature (Bizuneh 2014; Kalenga Saka and Msonthi 1994; Tsomele et al. 2021). Forest mahogany had a lower protein content (8.6%) compared with Natal mahogany, despite their taxonomic relatedness, which likely reflects variation in environmental conditions and genetic factors. Stamvrug exhibited the lowest protein content (5.5%), indicating a limited contribution to dietary protein intake compared with the other species.

The amino acid composition of the indigenous seed flours varied significantly (*p* < 0.05) (Table 2). The nonessential to essential amino acid (NEAA:EAA) ratios ranged from 1.8 to 2.7, indicating differences in the balance between nonessential and essential amino acids. Lower ratios observed in Lowveld chestnut and stamvrug (1.8) suggest a comparatively higher contribution of essential amino acids, whereas the higher ratio in marula (2.7) reflects a greater relative proportion of nonessential amino acids.

Considerable variation was evident in the essential amino acid profiles. Lowveld chestnut contained the highest lysine (7.0 g/100 g protein) and histidine (6.9 g/100 g protein), both of which are typically limiting in cereal‐based proteins. Available data for Lowveld chestnut are largely restricted to free amino acid profiles (Regnier et al. 2017), which are not directly comparable to the total amino acid composition determined in the present study. Marula contained 5.4 g/100 g protein leucine, which is within the reported range of 4.3–7.4 g/100 g protein (Muhammad et al. 2011; Magaia and Skog 2017), and 5.2 g/100 g protein valine, consistent with the literature value of approximately 5.0 g/100 g protein (Magaia and Skog 2017). For the mahogany species, valine ranged from 6.7 to 7.4 g/100 g protein and isoleucine from 5.6 to 5.7 g/100 g protein. These values are lower than those reported by Tsomele et al. 2023), who found higher valine (9.9 g/100 g) and isoleucine (8.8–9.2 g/100 g) concentrations. Stamvrug was characterized by the highest threonine (5.7 g/100 g protein) and leucine (6.3 g/100 g protein) contents among the samples, although comparable literature data for this species were not available.

Amino acid score (AAS) values for adults are discussed here to facilitate comparison of protein quality, while values for other age groups are provided in Table S2. Leucine was identified as the limiting amino acid in Lowveld chestnut (AAS  =  0.74), whereas lysine was limiting in marula (AAS  =  0.52), forest mahogany (AAS  =  0.90), Natal mahogany (AAS  =  0.90), and stamvrug (AAS  =  0.85). These findings indicate variation in protein quality among the flours, with Lowveld chestnut characterized by a favorable lysine profile but constrained by leucine, and marula by high total protein but comparatively lower lysine adequacy. Given that wheat flour is typically deficient in lysine and histidine (Southern African Grain Laboratory NPC 2025), incorporation of Lowveld chestnut into composite formulations may enhance lysine availability, whereas marula may contribute primarily to overall protein enrichment rather than amino acid balance.

#### 3.3.3 Lipid Content

The lipid content of the indigenous seed flours differed significantly among species (*p* < 0.05) (Table 2). Forest mahogany and Natal mahogany contained the highest lipid concentrations, at 41.8% and 40.8%, respectively, followed by marula at 34.5%. In contrast, Lowveld chestnut and stamvrug had substantially lower lipid contents of 7.5% and 4.9%, respectively. The high lipid levels observed for marula and the two mahogany species are consistent with previous reports describing these seeds as oil‐rich, although reported values vary with seed origin and extraction method (Kamanula et al. 2022; Mabaso et al. 2025). The lipid content of Lowveld chestnut was comparable to the 7.59% reported for raw nuts by Regnier et al. (2017), although higher values have been reported after roasting. The implications of these lipid differences for dough rheology and bread quality are discussed in later Sections 3.5 and 3.6.

Although the nutrient composition of the baked breads was not analytically determined in the present study, estimated protein and fat contents for the composite bread formulations were calculated from the measured flour composition and formulation ratios, with adjustment for baking loss, and are provided in Table S4. These calculated values indicate the expected compositional trends, but direct analysis of the final baked products will be required in future work to confirm nutrient retention and support labelling claims.

#### 3.3.4 Ash and Mineral Profile

The ash content and mineral composition of the indigenous flours varied significantly (*p* < 0.05) (Table 3). Forest mahogany had the highest ash content (4.19%), followed by marula (3.68%) and Lowveld chestnut (3.22%), while stamvrug had the lowest value (1.49%). Lowveld chestnut was characterized by moderate major mineral levels and the highest zinc concentration (44.50 mg/kg) among the samples (*p* < 0.05). In comparison, Regnier et al. 2017) reported substantially higher concentrations of major minerals for this species, including phosphorus (2.80 vs. 0.19 g/kg), magnesium (1.50 vs. 0.10 g/kg), potassium (5.30 vs. 0.86 g/kg), and calcium (0.90 vs. 0.01 g/kg), whereas iron and manganese were of comparable magnitude, and zinc and copper values were higher in the present study.

**TABLE 3:** Mineral composition of selected indigenous seed flours (Lowveld chestnut, marula, forest mahogany, Natal mahogany, and stamvrug) (g/kg for major elements; mg/kg for trace elements) (*n* = 3).

	Lowveld chestnut	Marula	Forest mahogany	Natal mahogany	Stamvrug
Ash (%)	3.22 ^c^ ±0.03	3.68 ^b^ ±0.12	4.19 ^a^ ±0.41	2.97 ^c^ ±0.01	1.49 ^d^ ±0.01
Major elements (g/kg)					
Phosphorus (P)	0.19 ^b^ ±0.06	0.52 ^a^ ±0.03	0.10 ^bc^ ±0.10	0.16 ^bc^ ±0.11	0.05 ^c^ ±0.00
Potassium (K)	0.86 ^a^ ±0.25	0.59 ^a^ ±0.03	0.76 ^a^ ±0.63	0.84 ^a^ ±0.49	0.63 ^a^ ±0.04
Calcium (Ca)	0.01 ^a^ ±0.01	0.05 ^a^ ±0.04	0.22 ^a^ ±0.18	0.17 ^a^ ±0.10	0.00 ^a^ ±0.00
Magnesium (Mg)	0.10 ^b^ ±0.02	0.38 ^a^ ±0.03	0.08 ^b^ ±0.07	0.14 ^b^ ±0.08	0.06 ^b^ ±0.01
Sodium (Na)	0.01 ^a^ ±0.02	0.02 ^a^ ±0.01	0.02 ^a^ ±0.01	0.02 ^a^ ±0.02	0.00 ^a^ ±0.01
Trace elements (mg/kg)					
Zinc (Zn)	44.50 ^a^ ±12.16	39.59 ^ab^ ±0.37	13.62 ^c^ ±11.59	22.70 ^bc^ ±12.38	10.37 ^c^ ±0.62
Copper (Cu)	15.46 ^a^ ±4.99	16.89 ^a^ ±1.02	6.30 ^b^ ±5.32	8.35 ^b^ ±4.4	5.43 ^b^ ±0.44
Manganese (Mn)	18.92 ^a^ ±7.25	11.97 ^a^ ±0.63	11.29 ^a^ ±9.40	17.50 ^a^ ±8.60	6.70 ^a^ ±0.67
Iron (Fe)	27.74 ^a^ ±7.12	62.55 ^a^ ±12.16	27.04 ^a^ ±21.23	40.83 ^a^ ±21.28	25.44 ^a^ ±1.64

*Note*: Different letters in the same row indicate significant differences between samples by Fisher's protected least significant difference (LSD) test (*p* < 0.05) followed by ±SD.

Marula flour was distinguished by significantly higher phosphorus and magnesium contents (*p* < 0.05), while other macro‐ and trace minerals showed limited differentiation among species. Reported mineral values for marula vary widely in the literature, with both lower and substantially higher concentrations documented for key elements such as iron, phosphorus, and magnesium (Glew et al. 1997; Aganga and Mosase 2001), suggesting strong geographical and compositional variability. Forest and Natal mahogany flours were characterized by high ash contents and broadly similar mineral profiles, consistent with their close taxonomic relationship. Reported ash and mineral values for these species are generally consistent with the literature, which indicates ash contents of ∼3.4%–4.5% and variable mineral concentrations depending on seed origin (Bizuneh 2014; Saka and Msonthi 1994; Tsomele et al. 2021). Stamvrug flour had the lowest ash content (1.49%) and generally lower mineral concentrations than the other indigenous seeds. Published information on the mineral and trace element profile of stamvrug seed flour appears to be lacking, and the present results therefore provide baseline data for the species.

### 3.4 Phenolic Compounds

**FIGURE 2:**
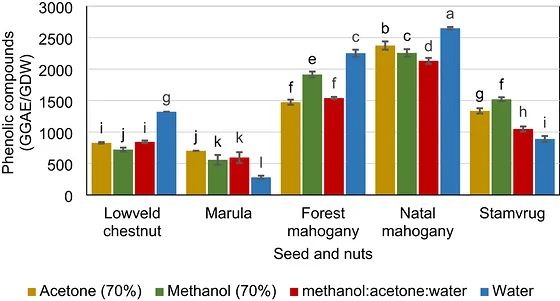
Total phenolic content of selected indigenous seed flours extracted using different solvents (µg gallic acid equivalents/g defatted sample). Bars with different letters are significantly different by Fisher's protected least significant difference (LSD) test (*p* < 0.05).

The comparative analysis of phenolic compound extraction revealed clear solvent‐dependent variability across seed types (Figure 2). Natal mahogany consistently exhibited the highest phenolic content across all solvents, with maximum recovery in water (2649.4 µg GAE/g), indicating the predominance of polar, water‐soluble phenolics. Forest mahogany and Lowveld chestnut showed similar extraction patterns, with peak values in water (2252 and 1322.4 µg GAE/g, respectively), although at lower levels than Natal mahogany. These trends suggest that phenolic compounds in these species are largely hydrophilic, which may enhance their antioxidant functionality in aqueous food systems. In contrast, marula exhibited the lowest phenolic content across all solvents, particularly in water (280.5 µg GAE/g), with acetone yielding the highest extraction efficiency. This pattern indicates a relatively lower abundance of water‐soluble phenolics and a greater contribution of less polar compounds.

Comparable solvent‐dependent behavior has been reported in both commercial oilseeds and indigenous African species (Naczk and Shahidi 2004; Shahidi and Ambigaipalan 2015). Studies on baobab, moringa, and other indigenous oilseeds have similarly demonstrated the presence of phenolic compounds of mixed polarity, contributing both to nutritional value and oxidative stability (Hilou et al. 2017; Dhlakama et al. 2022; Sabir et al. 2025). These compounds play a dual role in enhancing antioxidant potential and protecting lipid‐rich systems against oxidation during processing and storage. Overall, the findings highlight the importance of solvent selection in maximizing phenolic recovery and confirm that indigenous seeds represent valuable sources of phenolics with diverse polarity profiles, supporting their potential application in functional food systems.

### 3.5 Rheological Characterization Using Mixolab

Mixolab results demonstrated clear differences in rheological behavior between the wheat control and the composite flours (Table 4; Figure S1). The C1 torque, representing dough development and water absorption at 30°C, generally decreased with increasing substitution, indicating progressive weakening of the gluten network. This effect was most pronounced in marula, forest mahogany, and Natal mahogany composites, where C1 values were significantly lower than the control (1.32 Nm). In contrast, stamvrug flour exhibited an increase in C1 torque, reaching 1.78 Nm at 20% substitution despite its low protein content, suggesting that nonprotein components such as polysaccharides may have contributed to water binding and dough consistency rather than gluten formation (Fan et al. 2022).

**TABLE 4:** Mixolab rheological properties of composite dough made with indigenous seed flours (Lowveld chestnut, marula, forest mahogany, Natal mahogany, and stamvrug) (*n* = 3).

Dough sample	%	C1 (Nm)	C2 (Nm)	C3 (Nm)	C4 (Nm)	C5 (Nm)	Alpha	Beta	Gamma	Stability (minutes)
Control		1.32 ^b^ ±0.19	0.58 ^b^ ±0.10	1.83 ^b^ ±0.12	1.70 ^a^ ±0.15	2.55 ^bc^ ±0.23	−0.069 ^ef^ ±0.03	0.253 ^a^ ±0.07	−0.043 ^a^ ±0.02	8.48^ cd^ ±0.64
Lowveld chestnut	10%	0.98^ cd^ ±0.03	0.41 ^c^ ±0.02	1.43 ^c^ ±0.03	1.28 ^b^ ±0.01	2.13 ^de^ ±0.07	−0.024 ^bcd^ ±0.01	0.281 ^a^ ±0.02	−0.037 ^a^ ±0.01	9.96 ^ab^ ±0.24
	15%	0.87 ^cde^ ±0.02	0.36^ cd^ ±0.02	1.33^ cd^ ±0.02	1.20 ^b^ ±0.02	1.96 ^ef^ ±0.05	0.017 ^a^ ±0.00	0.243 ^a^ ±0.03	−0.041 ^a^ ±0.01	10.17 ^ab^ ±0.12
	20%	0.75 ^efg^ ±0.02	0.31 ^de^ ±0.02	1.20 ^de^ ±0.02	1.05 ^bcd^ ±0.03	1.81 ^fg^ ±0.02	−0.013 ^abc^ ±0.04	0.291 ^a^ ±0.02	−0.045 ^a^ ±0.02	10.23 ^ab^ ±0.13
Marula	10%	0.72 ^efgh^ ±0.02	0.26 ^ef^ ±0.04	1.15 ^ef^ ±0.06	1.03 ^bcd^ ±0.05	1.73 ^fg^ ±0.14	−0.013 ^abc^ ±0.01	0.291 ^a^ ±0.03	−0.017 ^a^ ±0.01	10.47 ^a^ ±0.15
	15%	0.59 ^ghi^ ±0.07	0.20 ^fg^ ±0.02	0.94 ^gh^ ±0.07	0.80 ^cde^ ±0.06	1.41 ^j^ ±0.11	−0.013 ^abc^ ±0.01	0.238 ^a^ ±0.02	−0.021 ^a^ ±0.01	10.52 ^a^ ±0.48
	20%	0.43 ^i^ ±0.03	0.17^ g^ ±0.01	0.83^ h^ ±0.02	0.68 ^de^ ±0.00	1.14 ^k^ ±0.01	0.010 ^ab^ ±0.01	0.279 ^a^ ±0.02	−0.027 ^a^ ±0.01	2.05^ g^ ±0.33
Forest mahogany	10%	0.87 ^cde^ ±0.01	0.36^ cd^ ±0.01	1.26 ^de^ ±0.03	1.20 ^b^ ±0.01	1.85 ^fg^ ±0.20	−0.057 ^de^ ±0.01	0.200 ^a^ ±0.08	0.002 ^a^ ±0.02	9.86 ^ab^ ±0.28
	15%	0.78 ^ef^ ±0.01	0.33 ^de^ ±0.01	1.15 ^ef^ ±0.03	0.93 ^bcde^ ±0.09	1.70 ^gh^ ±0.12	−0.018 ^abc^ ±0.02	0.254 ^a^ ±0.04	−0.134 ^a^ ±0.20	8.00 ^de^ ±0.08
	20%	0.72 ^efgh^ ±0.02	0.29 ^de^ ±0.02	1.03 ^fg^ ±0.03	0.70 ^cde^ ±0.02	1.45 ^hij^ ±0.10	−0.009 ^abc^ ±0.03	0.245 ^a^ ±0.02	−0.145 ^a^ ±0.12	7.08 ^ef^ ±0.08
Natal mahogany	10%	0.84 ^de^ ±0.12	0.32 ^de^ ±0.06	1.17 ^ef^ ±0.09	1.06 ^bc^ ±0.11	1.68 ^ghi^ ±0.16	−0.005 ^abc^ ±0.03	0.240 ^a^ ±0.01	−0.003 ^a^ ±0.02	9.11 ^bcd^ ±1.26
	15%	0.57 ^hi^	0.16^ g^	0.87 ^h^	0.76 ^cde^	1.23 ^jk^	−0.030^ cd^	0.356 ^a^	−0.028 ^a^	9.68 ^abc^
	20%	0.66 ^fgh^ ±0.02	0.20 ^fg^ ±0.01	0.90 ^gh^ ±0.01	0.61 ^e^ ±0.01	1.44 ^ij^ ±0.2	−0.033 ^cde^ ±0.02	0.248 ^a^ ±0.03	−0.097 ^a^ ±0.06	9.65 ^abc^ ±0.14
Stamvrug	10%	1.03 ^c^ ±0.07	0.52 ^b^ ±0.02	1.81 ^b^ ±0.02	1.68 ^a^ ±0.05	2.35^ cd^ ±0.06	−0.059 ^de^ ±0.04	0.288 ^a^ ±0.12	−0.038 ^a^ ±0.02	10.93 ^a^ ±0.56
	15%	1.25 ^b^ ±0.15	0.58 ^b^ ±0.07	2.05 ^a^ ±0.12	1.99 ^a^ ±0.11	2.60 ^b^ ±0.09	−0.007 ^abc^ ±0.01	0.190 ^a^ ±0.06	0.012 ^a^ ±0.04	9.83 ^ab^ ±0.65
	20%	1.78 ^a^ ±0.25	0.73 ^a^ ±0.11	1.92 ^ab^ ±0.24	1.79 ^a^ ±0.82	3.23 ^a^ ±0.38	−0.101 ^f^ ±0.02	0.206 ^a^ ±0.09	−0.034 ^a^ ±0.08	6.59 ^f^ ±2.32

*Note*: Different letters in the same column indicate significant differences between samples and percentage composite flours by Fisher's protected least significant difference (LSD) test (*p* < 0.05) followed by ±SD.

The C2 torque, associated with protein weakening under shear and heat, further highlighted differences in protein functionality. Marula substitution led to a marked decrease in C2, to 0.17 Nm at 20% inclusion, indicating reduced protein network stability. Conversely, stamvrug maintained a higher C2 value (0.73 Nm at 20%), suggesting the presence of heat‐stable structural components contributing to dough rigidity. Torque parameters associated with starch behavior (C3–C5) generally declined with increasing substitution, reflecting reduced starch gelatinization and paste stability. Marula produced the lowest C5 torque (1.14 Nm), indicating reduced retrogradation potential, whereas stamvrug exhibited the highest (3.23 Nm), indicating increased firmness.

Forest and Natal mahogany flours exhibited a characteristic reduction in torque during the heating phase, corresponding to a pronounced decline from C3 to C4. This behavior is indicative of reduced hot paste stability and structural breakdown of the starch–protein matrix during heating (Vézquez and Veira 2015; Szafrańska 2014). Such a decrease is commonly associated with increased susceptibility of gelatinized starch to degradation processes, which may involve endogenous α‐amylase activity. During heating, enzyme‐mediated starch hydrolysis can produce lower molecular weight dextrins, resulting in decreased viscosity and reduced torque (Codină et al. 2019). The observed patterns therefore suggest that the mahogany flours may contain active or residual enzymatic components contributing to starch breakdown during the high‐temperature phase. In addition, the presence of lipids in these flours may partially modulate this effect through the formation of amylose–lipid complexes, which can restrict starch swelling and limit enzymatic accessibility, thereby influencing the extent of structural degradation (Lee et al. 2024).

The results emphasize that rheological behavior in composite doughs is governed by protein functionality rather than protein content alone. Despite its high protein content (26.8%), marula produced the greatest reduction in torque because it lacks gluten‐forming proteins. Marula proteins are primarily globulins and albumins, which do not contribute to gluten network formation and therefore dilute the viscoelastic structure of wheat dough (Shewry and Halford 2001). This weakening effect was further exacerbated by the high lipid content of marula (34.5%), forest mahogany (41.8%), and Natal mahogany (40.8%). These lipids likely acted as lubricants, coating starch granules and protein structures, thereby reducing hydration, intermolecular interactions, and mechanical resistance during mixing (Khan and Shewry 2009). In contrast, the low lipid content of stamvrug (4.49%) resulted in higher torque values and a stiffer dough matrix, although with reduced stability at higher substitution levels.

Functionally, these rheological differences have important implications for bread quality. Lowveld chestnut maintained relatively high dough stability (up to 10.23 min) with moderate reductions in torque, indicating good tolerance to mechanical processing. Marula, although weakening the gluten network, significantly reduced retrogradation (low C5), suggesting potential for extending bread shelf life by delaying staling. Similar effects have been reported in composite systems where lipid‐rich ingredients reduce starch retrogradation and maintain product softness (Li et al. 2026). In contrast, stamvrug showed high torque but increased retrogradation and reduced stability, indicating a tendency toward dense, rigid bread with accelerated staling.

From a formulation perspective, there is a clear trade‐off between rheological performance and nutritional enhancement. Based on rheological behavior alone, Lowveld chestnut and marula at 10%–15% substitution provide the most stable dough systems. However, the bread compositional data (Table S4) indicate that substantial nutritional gains are only achieved at higher inclusion levels, particularly for marula, where protein content increases from 8.6% in the control to over 10% at 20% substitution alongside a marked increase in lipid content. In contrast, Lowveld chestnut contributes only modest increases in protein concentration but could enhance protein quality by increasing the concentration of essential amino acids deficient in wheat (e.g., lysine). Therefore, optimal substitution levels depend on the intended application: lower inclusion levels (10%–15%) favor processing stability and product quality, while higher levels (15%–20%) offer improved nutritional value but may require formulation adjustments, such as gluten supplementation or emulsifiers, to maintain dough functionality.

### 3.6 Baking Performance

#### 3.6.1 Color

**FIGURE 3:**
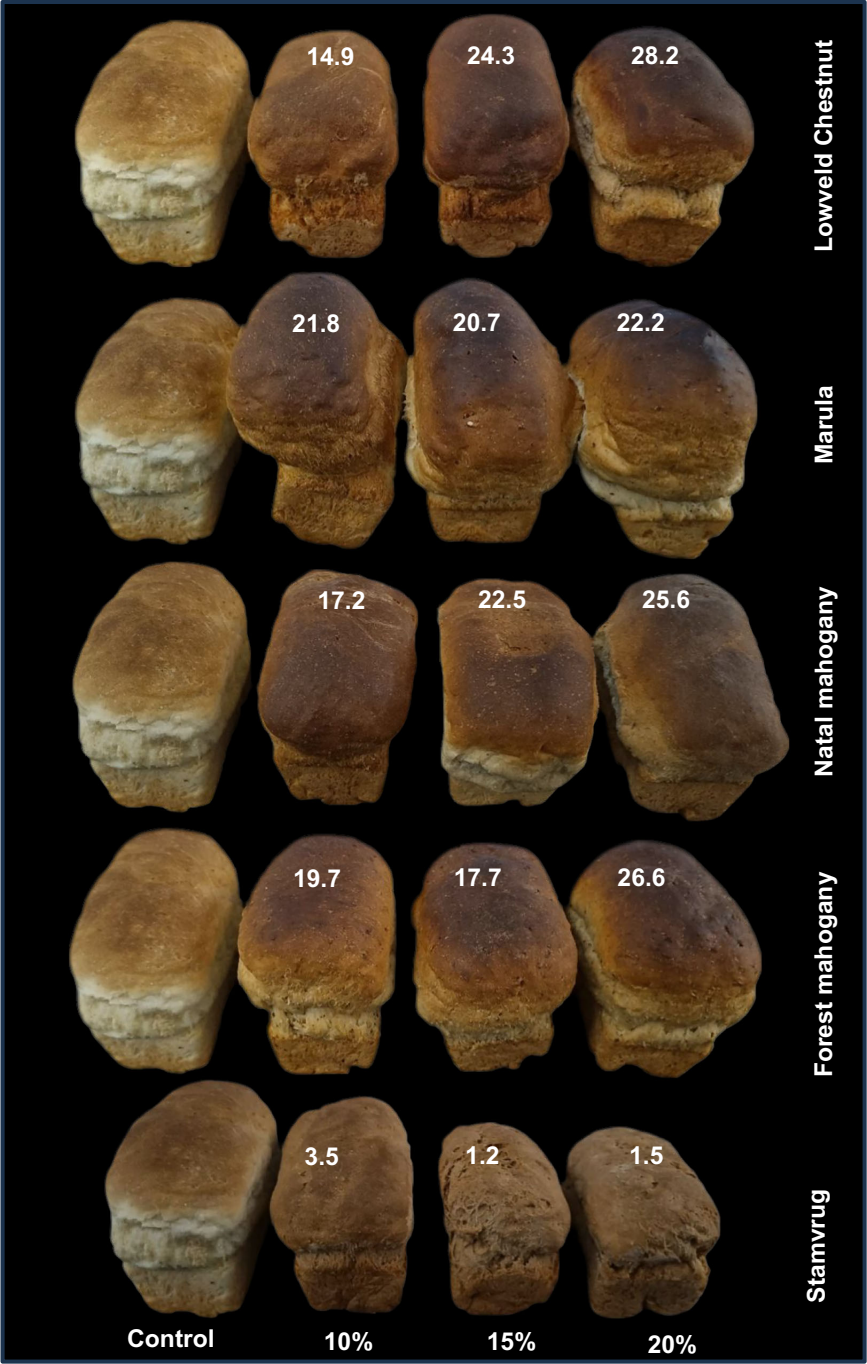
Photographic appearance of wheat control and composite bread loaves containing 10%, 15%, and 20% indigenous seed flours. Crust color differences (Δ*E*) relative to the control are indicated on the bread loaves.

The crust color (Figure 3) varied across treatments, with Δ*E* values indicating clearly perceptible differences from the wheat control. However, these differences did not follow a consistent or progressive trend with increasing inclusion. This suggests that, while flour composition contributes to crust appearance, color development at the dough surface is strongly influenced by processing conditions, particularly surface dehydration and Maillard and caramelization reactions during baking. These thermally driven reactions may partially mask compositional differences among the flours, resulting in greater variability and reduced sensitivity of crust color to substitution level compared with crumb color.

**FIGURE 4:**
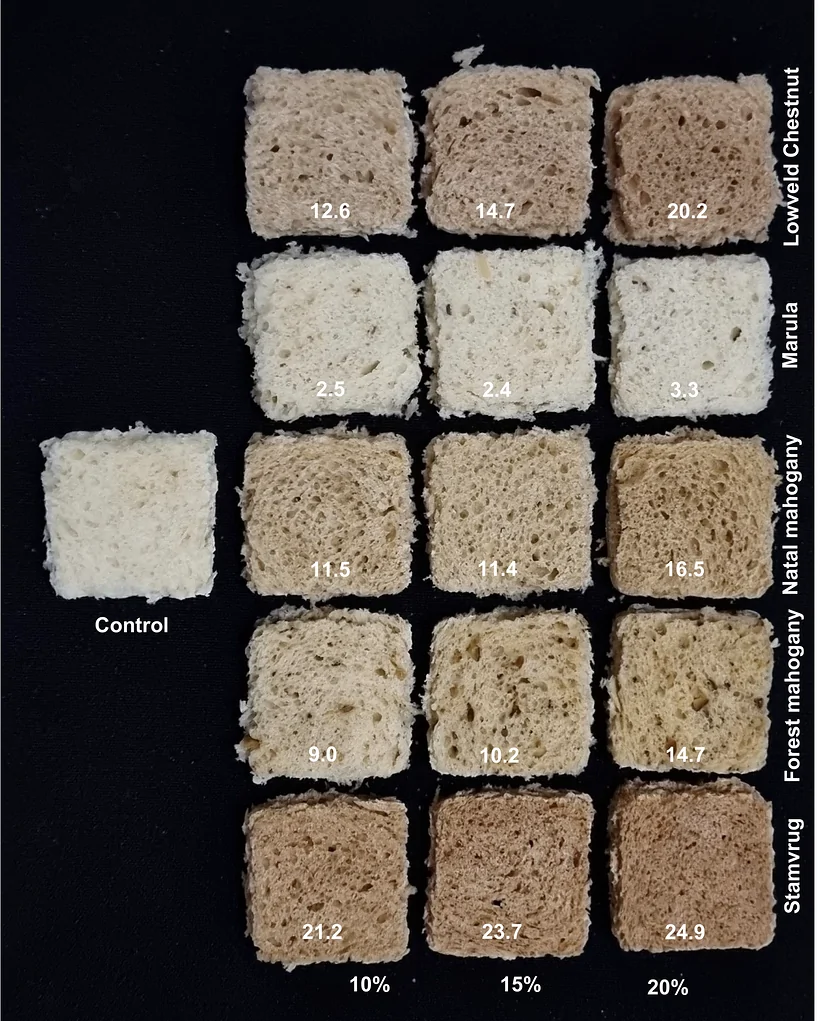
Photographic appearance of wheat control and composite breads containing 10%, 15%, and 20% indigenous seed flours. Crumb color differences (Δ*E*) are indicated on the images.

In contrast, crumb color was strongly and systematically influenced by both the type and level of indigenous flour inclusion (Figure 4; Table S3). Lightness (L*) decreased progressively with increasing substitution, indicating a clear darkening of the crumb. The wheat control exhibited a high L* value (74.9), while the most pronounced reductions were observed at 20% inclusion. Stamvrug and Lowveld chestnut composites showed the greatest darkening, with L* values of 52.4 and 56.1, respectively (Table S3). This resulted in substantial overall color differences (Δ*E*) relative to the control, particularly for stamvrug, where Δ*E* increased from 21.2 to 24.9 across the substitution range (Figure 4). In contrast, marula had a minimal effect on crumb color, with L* values comparable to the control and Δ*E* values ranging from 2.4 to 3.3, indicating limited visual deviation even at higher inclusion levels.

Changes in chromatic parameters further supported these observations. The a* values (red–green axis) increased across all composite breads, reflecting a shift toward more reddish‐brown hues, particularly in stamvrug and Lowveld chestnut at higher substitution levels. The b* values (yellow–blue axis) remained similar or slightly higher than the control in most blends. In contrast, marula showed a reduction in b*, indicating a shift away from yellow tones. These patterns were reflected in lower hue angles (H°) and higher chroma (C) values for the more pigmented flours, indicating increased color intensity and a shift toward red‐brown coloration (Table S3). Conversely, marula exhibited lower chroma values, consistent with its limited contribution to crumb color intensity. The relationship between instrumental color differences and consumer perception requires further investigation through sensory evaluation, as an instrumental color difference does not inherently imply reduced acceptability.

#### 3.6.2 Crumb Structure and Baking Loss

Baking loss and crumb structure were significantly influenced by both the type and level of indigenous seed flour inclusion, reflecting differences in water retention, gas cell stability, and dough matrix integrity during baking. Baking loss ranged from 12.8% in the 20% stamvrug composite to 18.9% in the 10% marula bread (Table 5). The higher baking loss observed in marula and mahogany composites may be attributed to weakened gluten networks and reduced water‐holding capacity, resulting in increased moisture loss during baking. In contrast, stamvrug composites, particularly at higher inclusion levels, exhibited lower baking loss, suggesting greater water retention within a more rigid dough matrix. Similar behavior has been reported in systems where reduced gluten functionality or structural cohesion affects moisture retention during thermal processing (Puhr and D'Appolonia 1992).

**TABLE 5:** Baking loss and C‑Cell crumb structure parameters of wheat control (W) and composite breads containing indigenous seed flours (Lowveld chestnut, marula, forest mahogany; Natal mahogany, stamvrug) (*n* = 3).

Bread sample	%	Baking loss (%)	Slice area (mm^2^)	No cells	Hole area (mm^2^)	Hole volume (mm^3^)	Wall thickness (mm)	Cell diameter (mm^2^)	Nonuniformity	Cell Volume (mm^3^)
Wheat		14.6 ^bcde^ ±1.76	4202 ^g^ ±313	3424 ^cd^ ±272	1.97^ cd^ ±0.88	45.19 ^bcd^ ±12.30	0.413 ^c^ ±0.006	1.65 ^ef^ ±0.06	4.36 ^cd^ ±2.17	5.24 ^e^ ±0.40
Lowveld chestnut	10%	13.9 ^cde^ ±1.54	4861 ^de^ ±578	3399^ cd^ ±199	2.09^ cd^ ±1.48	49.82 ^bcd^ ±21.53	0.420 ^bc^ ±0.010	1.96 ^c^ ±0.19	4.39 ^cd^ ±3.96	7.12 ^bcd^ ±0.87
	15%	13.1 ^de^ ±1.27	4967 ^cde^ ±119	3457 ^bcd^ ±25	2.36 ^bcd^ ±1.16	58.46 ^bcd^ ±16.28	0.427 ^abc^ ±0.006	1.89 ^cd^ ±0.09	4.83 ^cd^ ±3.10	7.03 ^bcd^ ±0.28
	20%	15.5 ^bc^ ±0.79	5079 ^bcd^ ±117	3482 ^bcd^ ±283	2.43 ^bcd^ ±2.04	57.72 ^bcd^ ±22.42	0.420 ^bc^ ±0.020	1.89 ^cd^ ±0.19	3.85 ^cd^ ±2.84	7.25 ^bcd^ ±1.01
Marula	10%	18.9 ^a^ ±1.27	4413 ^fg^ ±440	3289 ^cde^ ±621	5.21 ^ab^ ±1.58	75.51 ^abc^ ±18.33	0.430 ^abc^ ±0.010	2.34 ^a^ ±0.18	9.59 ^abc^ ±5.72	8.09 ^ab^ ±0.60
	15%	15.8 ^b^ ±2.30	5910 ^a^ ±231	3917 ^ab^ ±251	6.77 ^a^ ±1.59	98.95 ^a^ ±14.21	0.430 ^abc^ ±0.010	2.12 ^abc^ ±0.05	13.94 ^ab^ ±2.40	7.25 ^bcd^ ±0.38
	20%	15.2 ^bc^ ±2.04	6086 ^a^ ±228	4084 ^a^ ±312	6.90 ^a^ ±1.67	102.24 ^a^ ±14.72	0.430 ^abc^ ±0.017	2.27 ^ab^ ±0.22	16.95 ^a^ ±8.97	7.62 ^abcd^ ±0.94
Forest mahogany	10%	15.0 ^bc^ ±0.70	5324 ^bc^ ±278	3676 ^abc^ ±524	4.61 ^abc^ ±2.98	77.01 ^ab^ ±26.34	0.427 ^abc^ ±0.023	1.93 ^c^ ±0.13	8.11 ^bcd^ ±5.27	7.19 ^bcd^ ±0.60
	15%	15.2 ^bc^ ±0.59	5268 ^bcd^ ±263	3584 ^bcd^ ±29	3.38 ^bcd^ ±1.28	71.53 ^abc^ ±19.89	0.423 ^abc^ ±0.015	2.04 ^bc^ ±0.12	7.60 ^bcd^ ±6.37	7.58 ^abcd^ ±0.69
	20%	15.5 ^bc^ ±1.23	5409 ^b^ ±277	3619 ^abcd^ ±64	2.56 ^bcd^ ±1.15	58.02 ^bcd^ ±17.90	0.427 ^abc^ ±0.006	2.04 ^bc^ ±0.11	3.81 ^cd^ ±1.77	7.69 ^abc^ ±0.55
Natal mahogany	10%	14.2 ^bcde^ ±0.84	5356 ^bc^ ±69	3544 ^bcd^ ±307	2.20 ^cd^ ±0.93	55.92 ^bcd^ ±9.06	0.437 ^ab^ ±0.015	2.03 ^c^ ±0.19	4.88 ^cd^ ±3.21	7.73 ^abc^ ±0.86
	15%	14.1 ^bcde^ ±0.74	4868 ^de^ ±280	3289 ^cde^ ±136	1.40 ^d^ ±1.40	31.05 ^d^ ±27.62	0.427 ^abc^ ±0.021	1.92 ^cd^ ±0.20	2.46 ^cd^ ±1.83	6.96^ cd^ ±0.90
	20%	14.9 ^bc^ ±0.79	5121 ^bcd^ ±187	3175 ^de^ ±68	1.07 ^d^ ±1.85	23.05 ^d^ ±38.31	0.443 ^a^ ±0.006	2.12 ^abc^ ±0.10	1.68 ^d^ ±1.68	8.49 ^a^ ±0.36
Stamvrug	10%	15.2 ^bc^ ±0.72	4640 ^ef^ ±181	3500 ^bcd^ ±318	1.84 ^cd^ ±2.56	38.04 ^cd^ ±38.31	0.410 ^c^ ±0.010	1.69 ^de^ ±0.08	6.54 ^bcd^ ±8.66	6.57 ^d^ ±0.36
	15%	14.5 ^bcde^ ±0.44	3507 ^h^ ±19	3169 ^de^ ±108	0.90 ^d^ ±0.62	28.25 ^d^ ±13.29	0.383 ^d^ ±0.006	1.39 ^g^ ±0.05	1.48 ^d^ ±0.52	4.98 ^e^ ±0.36
	20%	12.8 ^e^ ±0.63	3260 ^h^ ±94	2922 ^e^ ±238	2.84 ^bcd^ ±2.82	45.92 ^bcd^ ±25.61	0.387 ^d ±^ 0.015	1.45 ^fg^ ±0.11	3.29 ^cd^ ±1.22	5.21 ^e^ ±0.61

*Note*: Different letters in the same column indicate significant differences between samples and percentage composite flours by Fisher's protected least significant difference (LSD) test (*p* < 0.05) followed by ±SD.

**FIGURE 5:**
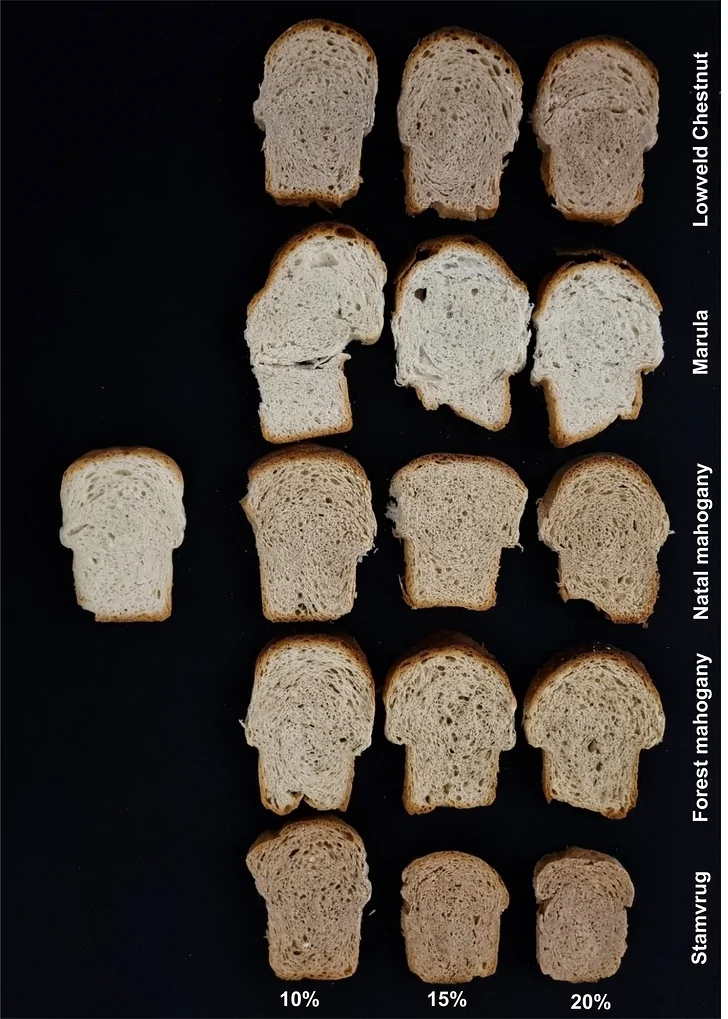
Crumb structure of bread slices (longitudinal section) of wheat control and composite breads containing 10%, 15%, and 20% of selected indigenous seed flours.

C‐Cell analysis provided detailed insight into crumb cellular architecture and loaf expansion (Table 5). Marula composites, particularly at 15% and 20% inclusion, exhibited significantly larger slice areas (5910 and 6086 mm^2^, respectively) compared with the wheat control (4202 mm^2^), indicating enhanced loaf expansion. This was accompanied by increased hole area and volume, reaching 6.90 and 102.24 mm^3^, respectively, at 20% substitution, as well as higher nonuniformity values. These parameters collectively indicate a coarse and open crumb structure characterized by large, irregular gas cells, as also evident in the crumb images (Figure 5). Such structures suggest that the weakened gluten network was unable to stabilize gas cells effectively, resulting in cell coalescence during proofing and baking and, in some cases, structural collapse (illustrated in Figure S2).

In contrast, stamvrug composites showed a progressive reduction in slice area and cell size with increasing substitution, with the 20% formulation exhibiting a reduced slice area (3260 mm^2^) and smaller cell diameter (1.45 mm), consistent with a dense and compact crumb structure. This is supported by the more uniform and tightly packed cell distribution observed in Figure 5 and aligns with the higher dough consistency (C1) and increased matrix rigidity observed in Mixolab analysis, which likely restricted gas expansion during proofing and baking.

Natal mahogany and Lowveld chestnut composites displayed intermediate structural characteristics, with more uniform crumb structures than marula but without the pronounced densification observed in stamvrug. The crumb images (Figure 5) show relatively stable and symmetrical cell distribution, suggesting a more balanced gas retention capacity and improved structural stability during baking.

These structural differences are consistent with previous findings that protein functionality and fiber content influence gas retention, loaf volume, and crumb architecture in composite breads (Renzetti et al. 2023; Miranda et al. 2023). Systems with reduced gluten functionality tend to produce larger and less stable gas cells, whereas more rigid matrices restrict expansion and produce denser crumb structures. In the present study, marula promoted loaf expansion at the expense of structural stability, while stamvrug limited expansion, resulting in a compact crumb. Overall, the C‐Cell analysis demonstrates that indigenous seed flours distinctly modify crumb architecture by shifting the balance between gas cell expansion and structural stabilization, with direct implications for loaf volume, crumb uniformity, and subsequent textural behavior.

#### 3.6.3 Crumb Firmness

**FIGURE 6:**
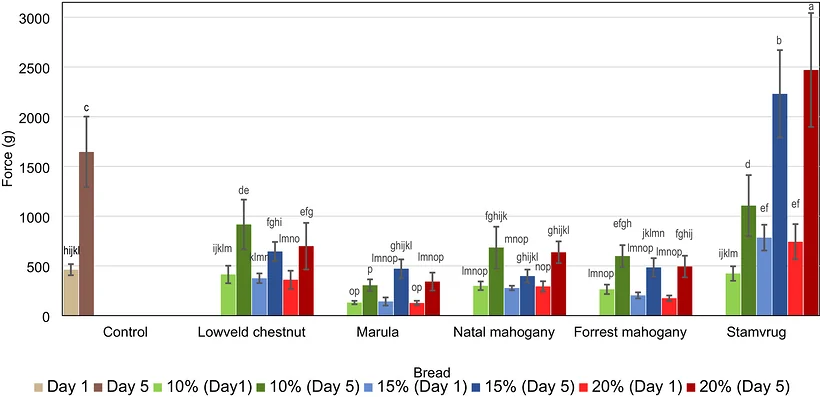
Firmness (force, g) of wheat control and composite breads at days 1 and 5 of storage. Values with different superscript letters are significantly different by Fisher's protected least significant difference (LSD) test (*p* < 0.05).

Crumb firmness measured at days 1 and 5 indicated pronounced differences in textural development and staling behavior among the composite loaves (Figure 6). The initial firmness of the wheat control was 462 g, increasing substantially to 1646 g after 5 days, reflecting typical firming associated with starch retrogradation. In contrast, marula and forest mahogany composites produced the softest crumbs at both time points, particularly at higher inclusion levels. At 20% substitution, marula and forest mahogany exhibited low initial firmness values of 127 and 175 g, respectively, and maintained significantly lower firmness after storage, with 20% marula reaching only 342 g at day 5. This sustained softness corresponds with the open crumb structures observed (Figure 5) and in the C‐Cell data (Table 5), where larger gas cells and higher void volumes contribute to reduced resistance to compression. These observations are also consistent with the Mixolab results, where marula and both mahogany flours exhibited low C5 torque values, indicative of reduced starch retrogradation. The high lipid content of these flours may have promoted amylose‐lipid complex formation and moderated amylopectin retrogradation and moisture redistribution during storage, thereby slowing crumb firming and preserving softness.

Lowveld chestnut and Natal mahogany composites displayed intermediate behavior, with lower firmness than the control but more structured crumb systems than marula. Their relatively uniform crumb architecture (Figure 5) suggests improved gas cell stability, which supports moderate softness while maintaining structural integrity. In contrast, stamvrug composites resulted in rapid firming, reaching 2230 and 2470 g for the 15% and 20% breads, respectively. This behavior aligns with the dense and compact crumb structure observed (Figure 5) and in the C‐Cell data (Table 5), where restricted gas cell expansion produced a tightly packed matrix with greater resistance to deformation.

## 4 Conclusions

The incorporation of indigenous South African seed flours (Lowveld chestnut, marula, Natal mahogany, forest mahogany, and stamvrug) into wheat bread significantly altered dough rheology, crumb structure and textural properties, with these effects closely associated with differences in flour composition. Marula flour, which had the highest protein content among the flours evaluated, contributed to protein and lipid enrichment and produced breads with softer crumbs during storage. Lowveld chestnut provided a favorable essential amino acid profile, particularly with respect to lysine, and maintained comparatively acceptable baking performance. In contrast, the inclusion of stamvrug flour produced a stiffer dough matrix, resulting in denser breads and higher crumb firmness after storage.

This study provides important baseline data on the nutritional, phenolic and microbiological characteristics of underutilized South African seed flours and demonstrates their potential application in composite bread systems. Although baking substantially reduced microbial loads, changes observed during storage emphasize the need to control raw material quality and postbaking handling to ensure product safety. Among the flours evaluated, marula and Lowveld chestnut offered the most favorable balance between nutritional enhancement and product functionality, demonstrating their potential use in improving the nutrient density of wheat‐based bread. Future work should include direct nutritional analysis of the final baked products and sensory evaluation to confirm nutrient retention, support labeling claims, and assess consumer acceptability.

## Author Contributions

**Karen de Jager**: conceptualization, investigation, Writing – original draft, methodology, visualization, formal analysis, data curation. **Wilma Augustyn**: conceptualization, methodology, supervision, writing – review and editing. **Thierry Regnier**: conceptualization, methodology, writing – review and editing, supervision. **Belinda Meiring**: conceptualization, methodology, writing – review and editing, supervision.

## Conflicts of Interest

The authors declare no conflicts of interest.

## Supporting information




**Supplementary Figures**: jfds71382‐sup‐0001‐Figures.docx

**Supplementary Tables**: jfds71382‐sup‐0002‐Tables.docx

## Data Availability

The data that support the findings of this study are available from the corresponding author upon reasonable request.
