# Supplementary material for: Nutritional Characterization of Selected South African Indigenous Seed Flours and Their Impact on Bread Baking Performance
Source: J Food Sci. 2026 Aug 22;91(8):e71382. doi: 10.1111/1750-3841.71382 (PMC13499083; doi:10.1111/1750-3841.71382)
Supplement: Supplementary file 1 [file JFDS-91-0-s001.docx]

Table S1: Microorganisms identified in indigenous seed flours and corresponding composite breads after 5 days of storage

| Plant species | Identified organisms in flour | Identified organisms in bread after 5 days |
| --- | --- | --- |
| Control |  | *Cladosporium halotolerans / Cladosporium cladosporioides*  *Penicillium citrinum** |
|  |  |  |
| Lowveld chestnut | *Exophiala sp.*  Hypocreales member  *Meira sp.* | *Aspergillus niger / Aspergillus tubingensis*  *Bacillus subtilis*  *Bacillus amyloliquefaciens / Bacillus velezensis / Bacillus nakamurai*  *Bacillus velezensis*  *Cladosporium tenuissimum / Cladosporium cladosporioides*  *Syncephalastrum sp. / Syncephalastrum breviphorum* |
|  |  |  |
| Marula | *Candida friedrichii*  *Candida membranifaciens*  *Herbaspirillum huttiense*  *Novospingobium capsulatum*  *Rhizopus stolonifera* | *Aspergillus nomiae*  *Aspergillus sydowii / Aspergillus versicolor*  *Penicillium citrinum**  *Penicillium commune / Penicillium crustosum**  *Syncephalastrum breviphorum* |
|  |  |  |
| Forest mahogany | *Aspergillus sydowii*  *Herbaspirillum huttiense* | *Bacillus amyloliquefaciens / Bacillus velezensis*  *Bacillus amyloliquefaciens / Bacillus velezensis / Bacillus nakamurai*  *Cladosporium pseudocladosporioides / Cladosporium cladosporioides*  *Penicillium sizovae / Penicillium dierckxii**  *Syncephalastrum breviphorum* |
|  |  |  |
| Natal mahogany | *Microbacterieum laevaniformans*  *Pseudomonas sp.*  *Rhizopos stolonifera* | *Alternaria sp.**  *Penicillium citrinum**  *Penicillium sizovae / Penicillium dierckxii**  *Talaromyces amestolkiae / Talaromyces purpureogenus* |
|  |  |  |
| Stamvrug | *Coniochaeta hoffmannii*  *Fusarium solani*  *Phyllobacterium myrsinacearum* | *Alternaria alternata / Alternaria tenuissima**  *Aspergillus pseudoglaucus / Aspergillus cibarius*  *Aspergillus sydowii*  *Cladosporium pseudocladosporioides / Cladosporium cladosporioides*  *Penicillium citrinum** |

* Potentially harmful organisms for human health

Table S2: Amino acid scores of wheat and Lowveld chestnut, marula, forest mahogany, Natal mahogany and stamvrug seed flours based on FAO/WHO (2007) reference patterns for different age groups

| FAO/WHO (2007) reference pattern | Wheat | Lowveld  chestnut | Marula | Forest  mahogany | Natal  mahogany | Stamvrug |
| --- | --- | --- | --- | --- | --- | --- |
| Histidine | | | | | | |
| Infants | 1.11 | 3.29 | 1.30 | 1.39 | 2.56 | 1.68 |
| Children 1–3 | 1.29 | 3.45 | 1.37 | 1.46 | 2.69 | 1.76 |
| Children 3–10 | 1.46 | 4.31 | 1.71 | 1.83 | 3.36 | 2.20 |
| Adults | 1.55 | 4.60 | 1.82 | 1.95 | 3.59 | 2.35 |
| Valine | | | | | | |
| Infants | 0.95 | 0.81 | 0.95 | 1.22 | 1.35 | 1.08 |
| Children 1–3 | 1.28 | 1.04 | 1.21 | 1.57 | 1.72 | 1.38 |
| Children 3–10 | 2.28 | 1.12 | 1.31 | 1.68 | 1.85 | 1.49 |
| Adults | 3.15 | 1.15 | 1.34 | 1.73 | 1.90 | 1.53 |
| Leucine | | | | | | |
| Infants | 1.20 | 0.45 | 0.56 | 0.63 | 0.64 | 0.65 |
| Children 1–3 | 1.25 | 0.66* | 0.82 | 0.91 | 0.94 | 0.95 |
| Children 3–10 | 1.58 | 0.71* | 0.88 | 0.99 | 1.02 | 1.03 |
| Adults | 4.16 | 0.74* | 0.91 | 1.02 | 1.05 | 1.06 |
| Isoleucine | | | | | | |
| Infants | 1.06 | 0.55 | 0.76 | 1.03 | 1.03 | 0.71 |
| Children 1–3 | 1.36 | 0.95 | 1.30 | 1.78 | 1.77 | 1.21 |
| Children 3–10 | 1.48 | 1.02 | 1.38 | 1.89 | 1.89 | 1.30 |
| Adults | 2.61 | 1.02 | 1.38 | 1.89 | 1.89 | 1.30 |
| Lysine | | | | | | |
| Infants | 0.42* | 1.01 | 0.34* | 0.59* | 0.59* | 0.55 |
| Children 1–3 | 0.58* | 1.22 | 0.41* | 0.71* | 0.72* | 0.67* |
| Children 3–10 | 0.65* | 1.45 | 0.49* | 0.84* | 0.85* | 0.80* |
| Adults | 0.93* | 1.55 | 0.52* | 0.90* | 0.91* | 0.85* |
| Methionine & Cysteine | | | | | | |
| Infants | 1.85 | 0.79 | 2.37 | 1.17 | 1.52 | 1.55 |
| Children 1–3 | 2.27 | 0.96 | 2.90 | 1.43 | 1.85 | 1.90 |
| Children 3–10 | 2.78 | 1.13 | 3.40 | 1.68 | 2.18 | 2.23 |
| Adults | 3.85 | 1.18 | 3.56 | 1.76 | 2.27 | 2.33 |
| Tyrosine & Phenylalanine | | | | | | |
| Infants | 1.85 | 0.44* | 0.58 | 0.62 | 0.62 | 0.54* |
| Children 1–3 | 2.27 | 0.79 | 1.06 | 1.12 | 1.12 | 0.98 |
| Children 3–10 | 2.33 | 1.01 | 1.34 | 1.42 | 1.42 | 1.24 |
| Adults | 4.05 | 1.09 | 1.45 | 1.54 | 1.53 | 1.34 |
| Threonine | | | | | | |
| Infants | 0.77 | 0.84 | 0.55 | 0.80 | 0.79 | 1.30 |
| Children 1–3 | 1.22 | 1.19 | 0.79 | 1.13 | 1.13 | 1.84 |
| Children 3–10 | 1.32 | 1.48 | 0.97 | 1.40 | 1.40 | 2.28 |
| Adults | 2.20 | 1.61 | 1.06 | 1.52 | 1.52 | 2.48 |

* Limiting amino acids

Table S3: Crumb colour of wheat and composite breads containing 10%, 15%, and 20% indigenous seed flours (n=3)

|  | 0% | 10% | 15% | 20% |
| --- | --- | --- | --- | --- |
| L* | | | | |
| Wheat flour | 74.9 ^a^ ±.57 |  |  |  |
| Lowveld chestnut |  | 63.4 ^cd^ ±1.65 | 61.7 ^de^ ±2.76 | 56.1 ^f^ ±0.31 |
| Marula |  | 72.9 ^a^ ±0.75 | 73.7 ^a^ ±0.66 | 73.2 ^a^ ±1.01 |
| Forrest mahogany |  | 66.7 ^b^ ±0.26 | 65.4 ^bc^ ±0.45 | 61.4 ^de^ ±0.42 |
| Natal mahogany |  | 64.7 ^bc^ ±0.40 | 64.3 ^c^ ±0.28 | 60.1 ^e^ ±0.35 |
| Stamvrug |  | 55.9 ^f^ ±0.00 | 53.9 ^fg^ ±1.34 | 52.4 ^g^ ±1.32 |
| a* | | | | |
| Wheat flour | 0.3 ^g^ ±0.21 |  |  |  |
| Lowveld chestnut |  | 5.1 ^d^ ±0.28 | 6.4 ^c^ ±0.05 | 7.5 ^b^ ±0.33 |
| Marula |  | 0.7 ^g^ ±0.38 | 0.4 ^g^ ±0.31 | 0.5 ^g^ ±0.75 |
| Forrest mahogany |  | 2.9 ^f^ ±0.19 | 2.7 ^f^ ±0.33 | 4.0 ^e^ ±0.47 |
| Natal mahogany |  | 4.2 ^e^ ±0.47 | 3.3 ^f^ ±0.09 | 5.8 ^cd^ ±0.02 |
| Stamvrug |  | 8.1 ^b^ ±0.00 | 9.7 ^a^ ±0.33 | 9.9 ^a^ ±0.31 |
| b* | | | | |
| Wheat flour | 17.1 ^g^ ±0.35 |  |  |  |
| Lowveld chestnut |  | 17.9 ^fg^ ±0.38 | 18.5 ^f^ ±0.07 | 18.4 ^f^ ±0.09 |
| Marula |  | 15.8 ^h^ ±0.09 | 15.0 ^hi^ ±0.26 | 14.3 ^i^ ±0.33 |
| Forrest mahogany |  | 19.7 ^e^ ±0.61 | 19.8 ^de^ ±0.31 | 21.3 ^bc^ ±0.24 |
| Natal mahogany |  | 20.7 ^cd^ ±0.14 | 20.0 ^de^ ±0.19 | 21.8 ^b^ ±1.01 |
| Stamvrug |  | 21.8 ^b^ ±0.75 | 22.8 ^a^ ±0.40 | 21.8 ^b^ ±0.33 |
| Hue | | | | |
| Wheat flour | 89.2 ^a^ ±0.26 |  |  |  |
| Lowveld chestnut |  | 74.2 ^e^ ±0.40 | 70.9 ^f^ ±0.61 | 67.9 ^gh^ ±2.17 |
| Marula |  | 87.4 ^a^ ±0.21 | 88.6 ^a^ ±0.26 | 87.9 ^a^ ±0.26 |
| Forrest mahogany |  | 81.6 ^b^ ±0.07 | 82.2 ^b^ ±0.28 | 79.4 ^cd^ ±1.81 |
| Natal mahogany |  | 78.6 ^d^ ±1.84 | 80.8 ^bc^ ±1.27 | 75.2 ^e^ ±0.42 |
| Stamvrug |  | 69.7 ^fg^ ±0.73 | 67.0 ^h^ ±1.06 | 65.8 ^h^ ±0.83 |
| Chroma | | | | |
| Wheat flour | 17.1 ^i^ ±0.45 |  |  |  |
| Lowveld chestnut |  | 18.7 ^h^ ±0.47 | 19.6 ^gh^ ±0.17 | 19.9 ^g^ ±0.05 |
| Marula |  | 15.9 ^j^ ±0.21 | 15.0 ^jk^ ±0.21 | 14.4 ^k^ ±0.31 |
| Forrest mahogany |  | 19.9 ^g^ ±0.61 | 20.0 ^g^ ±0.31 | 21.7 ^de^ ±0.24 |
| Natal mahogany |  | 21.1 ^ef^ ±0.16 | 20.3 ^fg^ ±0.21 | 22.6 ^cd^ ±0.87 |
| Stamvrug |  | 23.3 ^bc^ ±0.33 | 24.7 ^a^ ±0.50 | 23.9 ^ab^ ±1.04 |

Note: Different letters indicate significant differences between samples and percentage composite flours by Fisher's protected least significant difference (LSD) test (p < 0.05) followed by ±SD.

Table S4: Calculated^*^ protein and lipid composition (%) of control and composite breads after baking

| **Bread sample** | **Substitution (%)** | **Protein (%)** | **Lipid (%)** |
| --- | --- | --- | --- |
| Control (wheat) | 0 | 8.6 | 2.6 |
|  |  |  |  |
| Lowveld chestnut | 10 | 8.7 | 3.0 |
|  | 15 | 8.8 | 3.3 |
|  | 20 | 8.9 | 3.5 |
|  |  |  |  |
| Marula | 10 | 9.6 | 4.6 |
|  | 15 | 9.9 | 5.6 |
|  | 20 | 10.2 | 6.5 |
|  |  |  |  |
| Forest mahogany | 10 | 8.5 | 4.9 |
|  | 15 | 8.4 | 6.0 |
|  | 20 | 8.3 | 7.1 |
|  |  |  |  |
| Natal mahogany | 10 | 8.8 | 4.7 |
|  | 15 | 8.9 | 5.9 |
|  | 20 | 9.1 | 6.9 |
|  |  |  |  |
| Stamvrug | 10 | 8.3 | 2.9 |
|  | 15 | 8.1 | 3.0 |
|  | 20 | 7.9 | 3.2 |

* Protein and lipid contents were calculated by ingredient mass balance, assuming baking loss reflects moisture evaporation only.
