# Supplementary material for: Nutritional Characterization of Selected South African Indigenous Seed Flours and Their Impact on Bread Baking Performance
Source: J Food Sci. 2026 Aug 22;91(8):e71382. doi: 10.1111/1750-3841.71382 (PMC13499083; doi:10.1111/1750-3841.71382)
Supplement: Supplementary file 2 [file JFDS-91-0-s002.docx]

Torque (Nm)

Temperature (ºC)

|  | Wheat | LC10% | LC15% | LC20% | Dough temperature (°C) |
| --- | --- | --- | --- | --- | --- |
| 1 | 0 | 0.003 | 0 | 0 | 26.2 |
| 2 | 0.001 | 0 | 0 | 0 | 26.2 |
| 3 | 0.022 | 0.019 | 0.023 | 0.003 | 26.2 |
| 4 | 0.072 | 0.062 | 0.076 | 0.037 | 26.2 |
| 5 | 0.101 | 0.103 | 0.112 | 0.058 | 26.2 |
| 6 | 0.143 | 0.123 | 0.126 | 0.082 | 26.2 |
| 7 | 0.166 | 0.128 | 0.146 | 0.109 | 26.2 |
| 8 | 0.212 | 0.155 | 0.165 | 0.141 | 26.1 |
| 9 | 0.318 | 0.208 | 0.181 | 0.143 | 26.2 |
| 10 | 0.33 | 0.238 | 0.22 | 0.16 | 26.3 |
| 11 | 0.411 | 0.265 | 0.28 | 0.213 | 26.3 |
| 12 | 0.5 | 0.35 | 0.331 | 0.239 | 26.3 |
| 13 | 0.645 | 0.421 | 0.407 | 0.288 | 26.3 |
| 14 | 0.671 | 0.458 | 0.42 | 0.338 | 26.3 |
| 15 | 0.687 | 0.534 | 0.492 | 0.376 | 26.4 |
| 16 | 0.762 | 0.592 | 0.574 | 0.416 | 26.4 |
| 17 | 0.822 | 0.6 | 0.599 | 0.489 | 26.4 |
| 18 | 0.799 | 0.641 | 0.66 | 0.54 | 26.4 |
| 19 | 0.804 | 0.7 | 0.706 | 0.527 | 26.5 |
| 20 | 0.777 | 0.686 | 0.715 | 0.559 | 26.5 |
| 21 | 0.771 | 0.696 | 0.681 | 0.592 | 26.5 |
| 22 | 0.794 | 0.694 | 0.649 | 0.631 | 26.6 |
| 23 | 0.858 | 0.699 | 0.625 | 0.606 | 26.6 |
| 24 | 0.905 | 0.702 | 0.657 | 0.584 | 26.6 |
| 25 | 0.938 | 0.711 | 0.695 | 0.605 | 26.7 |
| 26 | 0.894 | 0.743 | 0.743 | 0.628 | 26.8 |
| 27 | 0.883 | 0.781 | 0.754 | 0.642 | 26.8 |
| 28 | 0.946 | 0.787 | 0.753 | 0.648 | 26.8 |
| 29 | 0.99 | 0.777 | 0.764 | 0.621 | 26.9 |
| 30 | 0.977 | 0.796 | 0.799 | 0.634 | 26.9 |
| 31 | 0.96 | 0.828 | 0.824 | 0.67 | 26.9 |
| 32 | 0.964 | 0.832 | 0.789 | 0.699 | 26.9 |
| 33 | 0.972 | 0.799 | 0.765 | 0.687 | 26.9 |
| 34 | 0.985 | 0.796 | 0.779 | 0.653 | 26.9 |
| 35 | 0.986 | 0.803 | 0.751 | 0.661 | 26.9 |
| 36 | 1.001 | 0.802 | 0.749 | 0.696 | 26.9 |
| 37 | 1.005 | 0.82 | 0.764 | 0.716 | 27 |
| 38 | 0.997 | 0.836 | 0.779 | 0.712 | 27 |
| 39 | 0.999 | 0.868 | 0.798 | 0.683 | 27 |
| 40 | 1.022 | 0.873 | 0.807 | 0.666 | 27 |
| 41 | 1.057 | 0.865 | 0.795 | 0.673 | 27.1 |
| 42 | 1.054 | 0.863 | 0.799 | 0.702 | 27.1 |
| 43 | 1.045 | 0.869 | 0.8 | 0.738 | 27.1 |
| 44 | 1.032 | 0.904 | 0.831 | 0.72 | 27.2 |
| 45 | 1.082 | 0.939 | 0.872 | 0.716 | 27.2 |
| 46 | 1.101 | 0.951 | 0.853 | 0.697 | 27.3 |
| 47 | 1.131 | 0.921 | 0.845 | 0.713 | 27.3 |
| 48 | 1.098 | 0.889 | 0.808 | 0.739 | 27.3 |
| 49 | 1.065 | 0.904 | 0.808 | 0.746 | 27.4 |
| 50 | 1.084 | 0.929 | 0.838 | 0.765 | 27.4 |
| 51 | 1.106 | 0.973 | 0.876 | 0.757 | 27.4 |
| 52 | 1.134 | 0.995 | 0.908 | 0.735 | 27.4 |
| 53 | 1.196 | 0.995 | 0.88 | 0.717 | 27.4 |
| 54 | 1.192 | 0.964 | 0.836 | 0.709 | 27.4 |
| 55 | 1.138 | 0.96 | 0.812 | 0.732 | 27.4 |
| 56 | 1.117 | 0.921 | 0.83 | 0.752 | 27.4 |
| 57 | 1.143 | 0.928 | 0.849 | 0.771 | 27.4 |
| 58 | 1.177 | 0.959 | 0.873 | 0.783 | 27.5 |
| 59 | 1.199 | 0.972 | 0.901 | 0.785 | 27.5 |
| 60 | 1.173 | 1.004 | 0.912 | 0.763 | 27.5 |
| 61 | 1.145 | 1 | 0.92 | 0.74 | 27.5 |
| 62 | 1.116 | 0.982 | 0.865 | 0.73 | 27.4 |
| 63 | 1.113 | 0.962 | 0.843 | 0.735 | 27.5 |
| 64 | 1.135 | 0.957 | 0.849 | 0.749 | 27.5 |
| 65 | 1.183 | 0.969 | 0.87 | 0.765 | 27.6 |
| 66 | 1.209 | 0.957 | 0.9 | 0.773 | 27.6 |
| 67 | 1.204 | 0.97 | 0.918 | 0.78 | 27.6 |
| 68 | 1.174 | 1.02 | 0.913 | 0.789 | 27.5 |
| 69 | 1.142 | 1.036 | 0.892 | 0.786 | 27.5 |
| 70 | 1.127 | 1.038 | 0.877 | 0.773 | 27.5 |
| 71 | 1.176 | 1.008 | 0.866 | 0.75 | 27.5 |
| 72 | 1.218 | 0.987 | 0.846 | 0.734 | 27.6 |
| 73 | 1.23 | 0.968 | 0.841 | 0.718 | 27.6 |
| 74 | 1.208 | 0.966 | 0.868 | 0.726 | 27.6 |
| 75 | 1.16 | 0.98 | 0.906 | 0.751 | 27.6 |
| 76 | 1.152 | 1.013 | 0.911 | 0.772 | 27.6 |
| 77 | 1.187 | 1.027 | 0.911 | 0.782 | 27.6 |
| 78 | 1.222 | 1.04 | 0.863 | 0.771 | 27.6 |
| 79 | 1.239 | 1.031 | 0.823 | 0.756 | 27.6 |
| 80 | 1.244 | 1.018 | 0.838 | 0.726 | 27.6 |
| 81 | 1.195 | 0.982 | 0.855 | 0.727 | 27.6 |
| 82 | 1.17 | 0.956 | 0.877 | 0.73 | 27.6 |
| 83 | 1.156 | 0.964 | 0.898 | 0.751 | 27.6 |
| 84 | 1.209 | 0.982 | 0.917 | 0.76 | 27.6 |
| 85 | 1.245 | 0.991 | 0.896 | 0.775 | 27.6 |
| 86 | 1.241 | 1.016 | 0.88 | 0.772 | 27.7 |
| 87 | 1.252 | 1.06 | 0.872 | 0.759 | 27.7 |
| 88 | 1.193 | 1.038 | 0.839 | 0.741 | 27.8 |
| 89 | 1.168 | 1.021 | 0.836 | 0.73 | 27.8 |
| 90 | 1.177 | 0.988 | 0.835 | 0.717 | 27.8 |
| 91 | 1.228 | 0.964 | 0.856 | 0.716 | 27.8 |
| 92 | 1.253 | 0.958 | 0.873 | 0.736 | 27.9 |
| 93 | 1.257 | 0.969 | 0.893 | 0.752 | 27.9 |
| 94 | 1.214 | 0.984 | 0.896 | 0.76 | 28 |
| 95 | 1.187 | 1.007 | 0.882 | 0.764 | 28 |
| 96 | 1.174 | 1.04 | 0.866 | 0.719 | 27.9 |
| 97 | 1.209 | 1.051 | 0.836 | 0.709 | 28 |
| 98 | 1.246 | 1.063 | 0.839 | 0.711 | 28 |
| 99 | 1.27 | 1.041 | 0.857 | 0.724 | 28 |
| 100 | 1.256 | 1.007 | 0.858 | 0.747 | 28.1 |
| 101 | 1.223 | 0.989 | 0.872 | 0.77 | 28.1 |
| 102 | 1.171 | 0.956 | 0.897 | 0.759 | 28.1 |
| 103 | 1.147 | 0.966 | 0.895 | 0.736 | 28 |
| 104 | 1.172 | 0.975 | 0.878 | 0.707 | 28 |
| 105 | 1.195 | 0.998 | 0.872 | 0.712 | 28 |
| 106 | 1.236 | 1.044 | 0.848 | 0.73 | 28 |
| 107 | 1.25 | 1.048 | 0.843 | 0.75 | 28.1 |
| 108 | 1.225 | 1.035 | 0.835 | 0.764 | 28 |
| 109 | 1.204 | 1.023 | 0.848 | 0.765 | 28.1 |
| 110 | 1.174 | 0.992 | 0.875 | 0.726 | 28 |
| 111 | 1.148 | 0.956 | 0.885 | 0.724 | 28 |
| 112 | 1.144 | 0.958 | 0.894 | 0.715 | 28 |
| 113 | 1.171 | 0.963 | 0.889 | 0.711 | 28 |
| 114 | 1.201 | 0.996 | 0.845 | 0.723 | 28.1 |
| 115 | 1.234 | 1.016 | 0.842 | 0.732 | 28.1 |
| 116 | 1.232 | 1.026 | 0.824 | 0.743 | 28.1 |
| 117 | 1.201 | 1.035 | 0.833 | 0.766 | 28 |
| 118 | 1.144 | 1.024 | 0.857 | 0.757 | 28.1 |
| 119 | 1.129 | 1.017 | 0.889 | 0.744 | 28.1 |
| 120 | 1.142 | 0.982 | 0.907 | 0.73 | 28.1 |
| 121 | 1.208 | 0.96 | 0.891 | 0.71 | 28.1 |
| 122 | 1.245 | 0.949 | 0.877 | 0.703 | 28.1 |
| 123 | 1.248 | 0.981 | 0.851 | 0.712 | 28.1 |
| 124 | 1.216 | 0.985 | 0.83 | 0.726 | 28.1 |
| 125 | 1.171 | 1.013 | 0.827 | 0.743 | 28.1 |
| 126 | 1.115 | 1.02 | 0.839 | 0.754 | 28.1 |
| 127 | 1.108 | 1.013 | 0.864 | 0.758 | 28.1 |
| 128 | 1.134 | 0.989 | 0.873 | 0.758 | 28.1 |
| 129 | 1.153 | 0.98 | 0.874 | 0.738 | 28.1 |
| 130 | 1.194 | 0.949 | 0.859 | 0.729 | 28.2 |
| 131 | 1.185 | 0.935 | 0.852 | 0.716 | 28.3 |
| 132 | 1.187 | 0.967 | 0.825 | 0.697 | 28.2 |
| 133 | 1.147 | 1.004 | 0.819 | 0.708 | 28.2 |
| 134 | 1.119 | 1.026 | 0.848 | 0.728 | 28.3 |
| 135 | 1.105 | 1.015 | 0.871 | 0.746 | 28.3 |
| 136 | 1.141 | 1.01 | 0.887 | 0.76 | 28.3 |
| 137 | 1.165 | 0.971 | 0.874 | 0.751 | 28.3 |
| 138 | 1.18 | 0.954 | 0.856 | 0.736 | 28.4 |
| 139 | 1.184 | 0.942 | 0.829 | 0.713 | 28.4 |
| 140 | 1.188 | 0.958 | 0.814 | 0.696 | 28.4 |
| 141 | 1.158 | 0.987 | 0.82 | 0.716 | 28.4 |
| 142 | 1.141 | 1.016 | 0.839 | 0.734 | 28.4 |
| 143 | 1.103 | 1.006 | 0.868 | 0.754 | 28.4 |
| 144 | 1.107 | 1.008 | 0.887 | 0.754 | 28.4 |
| 145 | 1.145 | 0.997 | 0.876 | 0.729 | 28.5 |
| 146 | 1.194 | 0.967 | 0.872 | 0.711 | 28.5 |
| 147 | 1.204 | 0.939 | 0.868 | 0.702 | 28.5 |
| 148 | 1.203 | 0.931 | 0.848 | 0.702 | 28.5 |
| 149 | 1.159 | 0.935 | 0.812 | 0.719 | 28.5 |
| 150 | 1.15 | 0.959 | 0.807 | 0.734 | 28.5 |
| 151 | 1.149 | 0.974 | 0.825 | 0.739 | 28.4 |
| 152 | 1.158 | 1.009 | 0.842 | 0.749 | 28.4 |
| 153 | 1.187 | 0.998 | 0.869 | 0.749 | 28.4 |
| 154 | 1.218 | 0.977 | 0.872 | 0.713 | 28.4 |
| 155 | 1.209 | 0.967 | 0.863 | 0.688 | 28.4 |
| 156 | 1.227 | 0.946 | 0.845 | 0.696 | 28.4 |
| 157 | 1.208 | 0.932 | 0.831 | 0.727 | 28.4 |
| 158 | 1.184 | 0.95 | 0.818 | 0.741 | 28.4 |
| 159 | 1.152 | 0.968 | 0.798 | 0.753 | 28.4 |
| 160 | 1.139 | 0.991 | 0.807 | 0.735 | 28.4 |
| 161 | 1.168 | 1.004 | 0.823 | 0.726 | 28.5 |
| 162 | 1.186 | 0.989 | 0.847 | 0.703 | 28.6 |
| 163 | 1.197 | 0.993 | 0.862 | 0.698 | 28.6 |
| 164 | 1.217 | 0.964 | 0.861 | 0.707 | 28.6 |
| 165 | 1.207 | 0.946 | 0.845 | 0.72 | 28.6 |
| 166 | 1.202 | 0.933 | 0.834 | 0.757 | 28.7 |
| 167 | 1.16 | 0.922 | 0.815 | 0.758 | 28.6 |
| 168 | 1.113 | 0.964 | 0.8 | 0.745 | 28.6 |
| 169 | 1.125 | 0.992 | 0.808 | 0.736 | 28.7 |
| 170 | 1.147 | 0.993 | 0.832 | 0.722 | 28.7 |
| 171 | 1.189 | 0.977 | 0.835 | 0.69 | 28.7 |
| 172 | 1.208 | 0.953 | 0.855 | 0.693 | 28.7 |
| 173 | 1.193 | 0.915 | 0.853 | 0.702 | 28.7 |
| 174 | 1.15 | 0.939 | 0.842 | 0.721 | 28.7 |
| 175 | 1.125 | 0.948 | 0.832 | 0.741 | 28.7 |
| 176 | 1.128 | 0.932 | 0.834 | 0.738 | 28.7 |
| 177 | 1.175 | 0.962 | 0.817 | 0.748 | 28.7 |
| 178 | 1.177 | 1.003 | 0.793 | 0.711 | 28.7 |
| 179 | 1.159 | 0.986 | 0.806 | 0.7 | 28.7 |
| 180 | 1.119 | 0.983 | 0.823 | 0.713 | 28.7 |
| 181 | 1.136 | 0.961 | 0.83 | 0.706 | 28.6 |
| 182 | 1.176 | 0.916 | 0.86 | 0.716 | 28.6 |
| 183 | 1.199 | 0.918 | 0.871 | 0.737 | 28.6 |
| 184 | 1.185 | 0.945 | 0.869 | 0.748 | 28.6 |
| 185 | 1.159 | 0.996 | 0.847 | 0.752 | 28.6 |
| 186 | 1.122 | 1.011 | 0.817 | 0.732 | 28.6 |
| 187 | 1.116 | 0.989 | 0.814 | 0.7 | 28.6 |
| 188 | 1.142 | 0.984 | 0.798 | 0.699 | 28.6 |
| 189 | 1.164 | 0.945 | 0.825 | 0.691 | 28.5 |
| 190 | 1.177 | 0.944 | 0.849 | 0.71 | 28.6 |
| 191 | 1.192 | 0.925 | 0.851 | 0.723 | 28.6 |
| 192 | 1.156 | 0.911 | 0.852 | 0.735 | 28.6 |
| 193 | 1.152 | 0.952 | 0.839 | 0.753 | 28.7 |
| 194 | 1.098 | 0.957 | 0.83 | 0.742 | 28.7 |
| 195 | 1.131 | 0.951 | 0.82 | 0.728 | 28.8 |
| 196 | 1.167 | 0.975 | 0.789 | 0.72 | 28.8 |
| 197 | 1.185 | 0.981 | 0.799 | 0.696 | 28.8 |
| 198 | 1.201 | 0.963 | 0.815 | 0.692 | 28.9 |
| 199 | 1.153 | 0.939 | 0.839 | 0.709 | 28.9 |
| 200 | 1.115 | 0.922 | 0.856 | 0.724 | 28.9 |

Torque (Nm)

Temperature ºC)

|  | Wheat | M10% | M15% | M20% | Dough temperature (°C) |
| --- | --- | --- | --- | --- | --- |
| 1 | 0 | 0 | 0 | 0 | 26.2 |
| 2 | 0.001 | 0.001 | 0 | 0 | 26.2 |
| 3 | 0.022 | 0.012 | 0.002 | 0.001 | 26.2 |
| 4 | 0.072 | 0.043 | 0.014 | 0.02 | 26.2 |
| 5 | 0.101 | 0.09 | 0.043 | 0.021 | 26.2 |
| 6 | 0.143 | 0.105 | 0.07 | 0.036 | 26.2 |
| 7 | 0.166 | 0.132 | 0.083 | 0.054 | 26.2 |
| 8 | 0.212 | 0.161 | 0.086 | 0.072 | 26.1 |
| 9 | 0.318 | 0.182 | 0.095 | 0.09 | 26.2 |
| 10 | 0.33 | 0.19 | 0.129 | 0.1 | 26.3 |
| 11 | 0.411 | 0.218 | 0.158 | 0.128 | 26.3 |
| 12 | 0.5 | 0.255 | 0.17 | 0.136 | 26.3 |
| 13 | 0.645 | 0.304 | 0.194 | 0.152 | 26.3 |
| 14 | 0.671 | 0.369 | 0.227 | 0.189 | 26.3 |
| 15 | 0.687 | 0.428 | 0.261 | 0.204 | 26.4 |
| 16 | 0.762 | 0.475 | 0.307 | 0.224 | 26.4 |
| 17 | 0.822 | 0.521 | 0.318 | 0.247 | 26.4 |
| 18 | 0.799 | 0.516 | 0.356 | 0.271 | 26.4 |
| 19 | 0.804 | 0.52 | 0.406 | 0.297 | 26.5 |
| 20 | 0.777 | 0.531 | 0.413 | 0.286 | 26.5 |
| 21 | 0.771 | 0.541 | 0.43 | 0.334 | 26.5 |
| 22 | 0.794 | 0.534 | 0.445 | 0.334 | 26.6 |
| 23 | 0.858 | 0.566 | 0.431 | 0.341 | 26.6 |
| 24 | 0.905 | 0.594 | 0.42 | 0.365 | 26.6 |
| 25 | 0.938 | 0.609 | 0.429 | 0.387 | 26.7 |
| 26 | 0.894 | 0.606 | 0.453 | 0.372 | 26.8 |
| 27 | 0.883 | 0.614 | 0.46 | 0.379 | 26.8 |
| 28 | 0.946 | 0.623 | 0.462 | 0.377 | 26.8 |
| 29 | 0.99 | 0.626 | 0.473 | 0.395 | 26.9 |
| 30 | 0.977 | 0.648 | 0.463 | 0.408 | 26.9 |
| 31 | 0.96 | 0.655 | 0.455 | 0.588 | 26.9 |
| 32 | 0.964 | 0.631 | 0.464 | 0.409 | 26.9 |
| 33 | 0.972 | 0.583 | 0.477 | 0.386 | 26.9 |
| 34 | 0.985 | 0.583 | 0.486 | 0.393 | 26.9 |
| 35 | 0.986 | 0.567 | 0.467 | 0.4 | 26.9 |
| 36 | 1.001 | 0.587 | 0.477 | 0.397 | 26.9 |
| 37 | 1.005 | 0.621 | 0.457 | 0.413 | 27 |
| 38 | 0.997 | 0.651 | 0.456 | 0.412 | 27 |
| 39 | 0.999 | 0.664 | 0.472 | 0.397 | 27 |
| 40 | 1.022 | 0.644 | 0.478 | 0.39 | 27 |
| 41 | 1.057 | 0.637 | 0.472 | 0.386 | 27.1 |
| 42 | 1.054 | 0.633 | 0.488 | 0.368 | 27.1 |
| 43 | 1.045 | 0.615 | 0.475 | 0.37 | 27.1 |
| 44 | 1.032 | 0.632 | 0.466 | 0.383 | 27.2 |
| 45 | 1.082 | 0.666 | 0.461 | 0.386 | 27.2 |
| 46 | 1.101 | 0.673 | 0.463 | 0.513 | 27.3 |
| 47 | 1.131 | 0.676 | 0.472 | 0.422 | 27.3 |
| 48 | 1.098 | 0.665 | 0.485 | 0.399 | 27.3 |
| 49 | 1.065 | 0.656 | 0.491 | 0.399 | 27.4 |
| 50 | 1.084 | 0.655 | 0.5 | 0.38 | 27.4 |
| 51 | 1.106 | 0.645 | 0.488 | 0.378 | 27.4 |
| 52 | 1.134 | 0.645 | 0.483 | 0.377 | 27.4 |
| 53 | 1.196 | 0.661 | 0.477 | 0.385 | 27.4 |
| 54 | 1.192 | 0.679 | 0.452 | 0.395 | 27.4 |
| 55 | 1.138 | 0.697 | 0.456 | 0.407 | 27.4 |
| 56 | 1.117 | 0.679 | 0.461 | 0.398 | 27.4 |
| 57 | 1.143 | 0.679 | 0.463 | 0.406 | 27.4 |
| 58 | 1.177 | 0.666 | 0.48 | 0.405 | 27.5 |
| 59 | 1.199 | 0.655 | 0.484 | 0.387 | 27.5 |
| 60 | 1.173 | 0.682 | 0.483 | 0.39 | 27.5 |
| 61 | 1.145 | 0.698 | 0.476 | 0.385 | 27.5 |
| 62 | 1.116 | 0.709 | 0.467 | 0.377 | 27.4 |
| 63 | 1.113 | 0.71 | 0.457 | 0.387 | 27.5 |
| 64 | 1.135 | 0.706 | 0.443 | 0.395 | 27.5 |
| 65 | 1.183 | 0.685 | 0.445 | 0.397 | 27.6 |
| 66 | 1.209 | 0.677 | 0.454 | 0.399 | 27.6 |
| 67 | 1.204 | 0.67 | 0.461 | 0.394 | 27.6 |
| 68 | 1.174 | 0.685 | 0.464 | 0.39 | 27.5 |
| 69 | 1.142 | 0.709 | 0.476 | 0.381 | 27.5 |
| 70 | 1.127 | 0.72 | 0.475 | 0.375 | 27.5 |
| 71 | 1.176 | 0.723 | 0.472 | 0.383 | 27.5 |
| 72 | 1.218 | 0.695 | 0.465 | 0.383 | 27.6 |
| 73 | 1.23 | 0.678 | 0.45 | 0.388 | 27.6 |
| 74 | 1.208 | 0.684 | 0.447 | 0.405 | 27.6 |
| 75 | 1.16 | 0.682 | 0.448 | 0.397 | 27.6 |
| 76 | 1.152 | 0.713 | 0.435 | 0.395 | 27.6 |
| 77 | 1.187 | 0.723 | 0.452 | 0.391 | 27.6 |
| 78 | 1.222 | 0.711 | 0.458 | 0.376 | 27.6 |
| 79 | 1.239 | 0.706 | 0.469 | 0.372 | 27.6 |
| 80 | 1.244 | 0.689 | 0.476 | 0.373 | 27.6 |
| 81 | 1.195 | 0.686 | 0.473 | 0.37 | 27.6 |
| 82 | 1.17 | 0.673 | 0.465 | 0.374 | 27.6 |
| 83 | 1.156 | 0.672 | 0.446 | 0.383 | 27.6 |
| 84 | 1.209 | 0.679 | 0.442 | 0.385 | 27.6 |
| 85 | 1.245 | 0.699 | 0.437 | 0.389 | 27.6 |
| 86 | 1.241 | 0.72 | 0.444 | 0.394 | 27.7 |
| 87 | 1.252 | 0.729 | 0.447 | 0.397 | 27.7 |
| 88 | 1.193 | 0.718 | 0.453 | 0.393 | 27.8 |
| 89 | 1.168 | 0.698 | 0.458 | 0.381 | 27.8 |
| 90 | 1.177 | 0.689 | 0.466 | 0.368 | 27.8 |
| 91 | 1.228 | 0.678 | 0.466 | 0.364 | 27.8 |
| 92 | 1.253 | 0.675 | 0.46 | 0.366 | 27.9 |
| 93 | 1.257 | 0.695 | 0.46 | 0.375 | 27.9 |
| 94 | 1.214 | 0.713 | 0.449 | 0.385 | 28 |
| 95 | 1.187 | 0.715 | 0.431 | 0.388 | 28 |
| 96 | 1.174 | 0.72 | 0.433 | 0.387 | 27.9 |
| 97 | 1.209 | 0.712 | 0.429 | 0.38 | 28 |
| 98 | 1.246 | 0.701 | 0.426 | 0.371 | 28 |
| 99 | 1.27 | 0.683 | 0.441 | 0.369 | 28 |
| 100 | 1.256 | 0.666 | 0.453 | 0.362 | 28.1 |
| 101 | 1.223 | 0.67 | 0.455 | 0.37 | 28.1 |
| 102 | 1.171 | 0.692 | 0.456 | 0.376 | 28.1 |
| 103 | 1.147 | 0.726 | 0.458 | 0.385 | 28 |
| 104 | 1.172 | 0.73 | 0.452 | 0.385 | 28 |
| 105 | 1.195 | 0.727 | 0.436 | 0.385 | 28 |
| 106 | 1.236 | 0.725 | 0.429 | 0.38 | 28 |
| 107 | 1.25 | 0.703 | 0.424 | 0.372 | 28.1 |
| 108 | 1.225 | 0.68 | 0.426 | 0.358 | 28 |
| 109 | 1.204 | 0.685 | 0.437 | 0.355 | 28.1 |
| 110 | 1.174 | 0.681 | 0.445 | 0.359 | 28 |
| 111 | 1.148 | 0.677 | 0.453 | 0.366 | 28 |
| 112 | 1.144 | 0.683 | 0.459 | 0.374 | 28 |
| 113 | 1.171 | 0.702 | 0.457 | 0.383 | 28 |
| 114 | 1.201 | 0.712 | 0.445 | 0.379 | 28.1 |
| 115 | 1.234 | 0.73 | 0.44 | 0.374 | 28.1 |
| 116 | 1.232 | 0.73 | 0.429 | 0.372 | 28.1 |
| 117 | 1.201 | 0.713 | 0.414 | 0.358 | 28 |
| 118 | 1.144 | 0.698 | 0.434 | 0.355 | 28.1 |
| 119 | 1.129 | 0.677 | 0.442 | 0.361 | 28.1 |
| 120 | 1.142 | 0.668 | 0.446 | 0.36 | 28.1 |
| 121 | 1.208 | 0.681 | 0.457 | 0.368 | 28.1 |
| 122 | 1.245 | 0.688 | 0.451 | 0.374 | 28.1 |
| 123 | 1.248 | 0.716 | 0.444 | 0.375 | 28.1 |
| 124 | 1.216 | 0.734 | 0.432 | 0.367 | 28.1 |
| 125 | 1.171 | 0.721 | 0.425 | 0.357 | 28.1 |
| 126 | 1.115 | 0.727 | 0.421 | 0.349 | 28.1 |
| 127 | 1.108 | 0.703 | 0.422 | 0.346 | 28.1 |
| 128 | 1.134 | 0.677 | 0.439 | 0.35 | 28.1 |
| 129 | 1.153 | 0.691 | 0.454 | 0.363 | 28.1 |
| 130 | 1.194 | 0.691 | 0.45 | 0.374 | 28.2 |
| 131 | 1.185 | 0.706 | 0.447 | 0.376 | 28.3 |
| 132 | 1.187 | 0.738 | 0.445 | 0.375 | 28.2 |
| 133 | 1.147 | 0.722 | 0.43 | 0.368 | 28.2 |
| 134 | 1.119 | 0.704 | 0.426 | 0.36 | 28.3 |
| 135 | 1.105 | 0.687 | 0.422 | 0.358 | 28.3 |
| 136 | 1.141 | 0.673 | 0.418 | 0.347 | 28.3 |
| 137 | 1.165 | 0.668 | 0.438 | 0.348 | 28.3 |
| 138 | 1.18 | 0.695 | 0.445 | 0.357 | 28.4 |
| 139 | 1.184 | 0.701 | 0.451 | 0.363 | 28.4 |
| 140 | 1.188 | 0.71 | 0.45 | 0.371 | 28.4 |
| 141 | 1.158 | 0.728 | 0.441 | 0.374 | 28.4 |
| 142 | 1.141 | 0.731 | 0.429 | 0.377 | 28.4 |
| 143 | 1.103 | 0.704 | 0.416 | 0.363 | 28.4 |
| 144 | 1.107 | 0.683 | 0.415 | 0.35 | 28.4 |
| 145 | 1.145 | 0.672 | 0.416 | 0.347 | 28.5 |
| 146 | 1.194 | 0.69 | 0.425 | 0.345 | 28.5 |
| 147 | 1.204 | 0.694 | 0.434 | 0.343 | 28.5 |
| 148 | 1.203 | 0.723 | 0.447 | 0.355 | 28.5 |
| 149 | 1.159 | 0.728 | 0.443 | 0.366 | 28.5 |
| 150 | 1.15 | 0.711 | 0.441 | 0.373 | 28.5 |
| 151 | 1.149 | 0.685 | 0.445 | 0.374 | 28.4 |
| 152 | 1.158 | 0.675 | 0.433 | 0.371 | 28.4 |
| 153 | 1.187 | 0.67 | 0.431 | 0.356 | 28.4 |
| 154 | 1.218 | 0.691 | 0.42 | 0.353 | 28.4 |
| 155 | 1.209 | 0.703 | 0.411 | 0.351 | 28.4 |
| 156 | 1.227 | 0.723 | 0.421 | 0.345 | 28.4 |
| 157 | 1.208 | 0.719 | 0.428 | 0.355 | 28.4 |
| 158 | 1.184 | 0.725 | 0.429 | 0.362 | 28.4 |
| 159 | 1.152 | 0.708 | 0.446 | 0.366 | 28.4 |
| 160 | 1.139 | 0.699 | 0.446 | 0.366 | 28.4 |
| 161 | 1.168 | 0.676 | 0.438 | 0.361 | 28.5 |
| 162 | 1.186 | 0.668 | 0.428 | 0.349 | 28.6 |
| 163 | 1.197 | 0.673 | 0.419 | 0.346 | 28.6 |
| 164 | 1.217 | 0.696 | 0.41 | 0.344 | 28.6 |
| 165 | 1.207 | 0.712 | 0.411 | 0.339 | 28.6 |
| 166 | 1.202 | 0.715 | 0.415 | 0.344 | 28.7 |
| 167 | 1.16 | 0.726 | 0.428 | 0.352 | 28.6 |
| 168 | 1.113 | 0.729 | 0.432 | 0.358 | 28.6 |
| 169 | 1.125 | 0.704 | 0.439 | 0.354 | 28.7 |
| 170 | 1.147 | 0.694 | 0.444 | 0.36 | 28.7 |
| 171 | 1.189 | 0.68 | 0.427 | 0.348 | 28.7 |
| 172 | 1.208 | 0.668 | 0.425 | 0.337 | 28.7 |
| 173 | 1.193 | 0.664 | 0.416 | 0.337 | 28.7 |
| 174 | 1.15 | 0.68 | 0.413 | 0.34 | 28.7 |
| 175 | 1.125 | 0.699 | 0.416 | 0.35 | 28.7 |
| 176 | 1.128 | 0.718 | 0.423 | 0.357 | 28.7 |
| 177 | 1.175 | 0.719 | 0.427 | 0.362 | 28.7 |
| 178 | 1.177 | 0.707 | 0.443 | 0.359 | 28.7 |
| 179 | 1.159 | 0.694 | 0.442 | 0.352 | 28.7 |
| 180 | 1.119 | 0.664 | 0.431 | 0.346 | 28.7 |
| 181 | 1.136 | 0.667 | 0.422 | 0.336 | 28.6 |
| 182 | 1.176 | 0.672 | 0.416 | 0.333 | 28.6 |
| 183 | 1.199 | 0.706 | 0.412 | 0.334 | 28.6 |
| 184 | 1.185 | 0.716 | 0.412 | 0.341 | 28.6 |
| 185 | 1.159 | 0.716 | 0.423 | 0.351 | 28.6 |
| 186 | 1.122 | 0.714 | 0.427 | 0.353 | 28.6 |
| 187 | 1.116 | 0.712 | 0.437 | 0.358 | 28.6 |
| 188 | 1.142 | 0.681 | 0.44 | 0.352 | 28.6 |
| 189 | 1.164 | 0.669 | 0.446 | 0.358 | 28.5 |
| 190 | 1.177 | 0.658 | 0.431 | 0.339 | 28.6 |
| 191 | 1.192 | 0.666 | 0.422 | 0.335 | 28.6 |
| 192 | 1.156 | 0.685 | 0.419 | 0.33 | 28.6 |
| 193 | 1.152 | 0.709 | 0.411 | 0.335 | 28.7 |
| 194 | 1.098 | 0.716 | 0.419 | 0.348 | 28.7 |
| 195 | 1.131 | 0.716 | 0.429 | 0.354 | 28.8 |
| 196 | 1.167 | 0.684 | 0.433 | 0.361 | 28.8 |
| 197 | 1.185 | 0.672 | 0.445 | 0.362 | 28.8 |
| 198 | 1.201 | 0.673 | 0.441 | 0.347 | 28.9 |
| 199 | 1.153 | 0.664 | 0.435 | 0.335 | 28.9 |
| 200 | 1.115 | 0.705 | 0.431 | 0.331 | 28.9 |

Torque (Nm)

Temperature (°C)

|  | Wheat | Td10% | Td15% | Td20% | Dough temperature (°C) |
| --- | --- | --- | --- | --- | --- |
| 1 | 0 | 0 | 0.001 | 0.004 | 26.2 |
| 2 | 0.001 | 0.001 | 0 | 0 | 26.2 |
| 3 | 0.022 | 0.027 | 0.011 | 0 | 26.2 |
| 4 | 0.072 | 0.073 | 0.045 | 0.004 | 26.2 |
| 5 | 0.101 | 0.092 | 0.048 | 0.04 | 26.2 |
| 6 | 0.143 | 0.105 | 0.09 | 0.061 | 26.2 |
| 7 | 0.166 | 0.128 | 0.098 | 0.074 | 26.2 |
| 8 | 0.212 | 0.18 | 0.116 | 0.105 | 26.1 |
| 9 | 0.318 | 0.19 | 0.138 | 0.132 | 26.2 |
| 10 | 0.33 | 0.2 | 0.187 | 0.155 | 26.3 |
| 11 | 0.411 | 0.268 | 0.211 | 0.175 | 26.3 |
| 12 | 0.5 | 0.341 | 0.252 | 0.181 | 26.3 |
| 13 | 0.645 | 0.409 | 0.283 | 0.227 | 26.3 |
| 14 | 0.671 | 0.463 | 0.307 | 0.235 | 26.3 |
| 15 | 0.687 | 0.523 | 0.355 | 0.272 | 26.4 |
| 16 | 0.762 | 0.547 | 0.401 | 0.318 | 26.4 |
| 17 | 0.822 | 0.615 | 0.434 | 0.384 | 26.4 |
| 18 | 0.799 | 0.641 | 0.462 | 0.417 | 26.4 |
| 19 | 0.804 | 0.664 | 0.504 | 0.437 | 26.5 |
| 20 | 0.777 | 0.678 | 0.529 | 0.435 | 26.5 |
| 21 | 0.771 | 0.651 | 0.555 | 0.44 | 26.5 |
| 22 | 0.794 | 0.651 | 0.567 | 0.434 | 26.6 |
| 23 | 0.858 | 0.7 | 0.552 | 0.459 | 26.6 |
| 24 | 0.905 | 0.725 | 0.554 | 0.468 | 26.6 |
| 25 | 0.938 | 0.678 | 0.58 | 0.467 | 26.7 |
| 26 | 0.894 | 0.696 | 0.567 | 0.482 | 26.8 |
| 27 | 0.883 | 0.71 | 0.568 | 0.474 | 26.8 |
| 28 | 0.946 | 0.713 | 0.573 | 0.499 | 26.8 |
| 29 | 0.99 | 0.758 | 0.553 | 0.51 | 26.9 |
| 30 | 0.977 | 0.785 | 0.575 | 0.508 | 26.9 |
| 31 | 0.96 | 0.831 | 0.597 | 0.502 | 26.9 |
| 32 | 0.964 | 0.836 | 0.588 | 0.491 | 26.9 |
| 33 | 0.972 | 0.784 | 0.58 | 0.48 | 26.9 |
| 34 | 0.985 | 0.76 | 0.558 | 0.485 | 26.9 |
| 35 | 0.986 | 0.755 | 0.56 | 0.502 | 26.9 |
| 36 | 1.001 | 0.774 | 0.571 | 0.509 | 26.9 |
| 37 | 1.005 | 0.791 | 0.578 | 0.506 | 27 |
| 38 | 0.997 | 0.786 | 0.599 | 0.506 | 27 |
| 39 | 0.999 | 0.793 | 0.593 | 0.497 | 27 |
| 40 | 1.022 | 0.807 | 0.603 | 0.486 | 27 |
| 41 | 1.057 | 0.814 | 0.605 | 0.491 | 27.1 |
| 42 | 1.054 | 0.802 | 0.591 | 0.496 | 27.1 |
| 43 | 1.045 | 0.786 | 0.59 | 0.519 | 27.1 |
| 44 | 1.032 | 0.803 | 0.589 | 0.523 | 27.2 |
| 45 | 1.082 | 0.817 | 0.605 | 0.516 | 27.2 |
| 46 | 1.101 | 0.831 | 0.622 | 0.521 | 27.3 |
| 47 | 1.131 | 0.851 | 0.639 | 0.52 | 27.3 |
| 48 | 1.098 | 0.82 | 0.622 | 0.511 | 27.3 |
| 49 | 1.065 | 0.812 | 0.613 | 0.507 | 27.4 |
| 50 | 1.084 | 0.8 | 0.602 | 0.513 | 27.4 |
| 51 | 1.106 | 0.785 | 0.606 | 0.52 | 27.4 |
| 52 | 1.134 | 0.815 | 0.635 | 0.53 | 27.4 |
| 53 | 1.196 | 0.866 | 0.643 | 0.523 | 27.4 |
| 54 | 1.192 | 0.876 | 0.644 | 0.514 | 27.4 |
| 55 | 1.138 | 0.838 | 0.628 | 0.509 | 27.4 |
| 56 | 1.117 | 0.831 | 0.613 | 0.523 | 27.4 |
| 57 | 1.143 | 0.814 | 0.622 | 0.539 | 27.4 |
| 58 | 1.177 | 0.806 | 0.633 | 0.544 | 27.5 |
| 59 | 1.199 | 0.8 | 0.639 | 0.536 | 27.5 |
| 60 | 1.173 | 0.828 | 0.663 | 0.523 | 27.5 |
| 61 | 1.145 | 0.855 | 0.653 | 0.516 | 27.5 |
| 62 | 1.116 | 0.87 | 0.637 | 0.506 | 27.4 |
| 63 | 1.113 | 0.831 | 0.62 | 0.519 | 27.5 |
| 64 | 1.135 | 0.824 | 0.621 | 0.533 | 27.5 |
| 65 | 1.183 | 0.793 | 0.621 | 0.549 | 27.6 |
| 66 | 1.209 | 0.795 | 0.635 | 0.551 | 27.6 |
| 67 | 1.204 | 0.822 | 0.653 | 0.54 | 27.6 |
| 68 | 1.174 | 0.842 | 0.658 | 0.531 | 27.5 |
| 69 | 1.142 | 0.865 | 0.655 | 0.523 | 27.5 |
| 70 | 1.127 | 0.841 | 0.664 | 0.519 | 27.5 |
| 71 | 1.176 | 0.827 | 0.654 | 0.531 | 27.5 |
| 72 | 1.218 | 0.806 | 0.639 | 0.542 | 27.6 |
| 73 | 1.23 | 0.778 | 0.636 | 0.555 | 27.6 |
| 74 | 1.208 | 0.811 | 0.634 | 0.554 | 27.6 |
| 75 | 1.16 | 0.83 | 0.618 | 0.548 | 27.6 |
| 76 | 1.152 | 0.869 | 0.64 | 0.536 | 27.6 |
| 77 | 1.187 | 0.862 | 0.643 | 0.528 | 27.6 |
| 78 | 1.222 | 0.825 | 0.654 | 0.527 | 27.6 |
| 79 | 1.239 | 0.803 | 0.672 | 0.532 | 27.6 |
| 80 | 1.244 | 0.814 | 0.661 | 0.547 | 27.6 |
| 81 | 1.195 | 0.824 | 0.643 | 0.555 | 27.6 |
| 82 | 1.17 | 0.85 | 0.622 | 0.56 | 27.6 |
| 83 | 1.156 | 0.878 | 0.626 | 0.562 | 27.6 |
| 84 | 1.209 | 0.859 | 0.64 | 0.548 | 27.6 |
| 85 | 1.245 | 0.832 | 0.656 | 0.535 | 27.6 |
| 86 | 1.241 | 0.816 | 0.67 | 0.53 | 27.7 |
| 87 | 1.252 | 0.811 | 0.665 | 0.528 | 27.7 |
| 88 | 1.193 | 0.826 | 0.66 | 0.536 | 27.8 |
| 89 | 1.168 | 0.854 | 0.651 | 0.552 | 27.8 |
| 90 | 1.177 | 0.869 | 0.647 | 0.566 | 27.8 |
| 91 | 1.228 | 0.873 | 0.626 | 0.561 | 27.8 |
| 92 | 1.253 | 0.833 | 0.622 | 0.549 | 27.9 |
| 93 | 1.257 | 0.825 | 0.635 | 0.536 | 27.9 |
| 94 | 1.214 | 0.806 | 0.643 | 0.533 | 28 |
| 95 | 1.187 | 0.797 | 0.651 | 0.541 | 28 |
| 96 | 1.174 | 0.837 | 0.674 | 0.556 | 27.9 |
| 97 | 1.209 | 0.852 | 0.665 | 0.566 | 28 |
| 98 | 1.246 | 0.864 | 0.661 | 0.57 | 28 |
| 99 | 1.27 | 0.861 | 0.65 | 0.57 | 28 |
| 100 | 1.256 | 0.848 | 0.631 | 0.554 | 28.1 |
| 101 | 1.223 | 0.818 | 0.623 | 0.553 | 28.1 |
| 102 | 1.171 | 0.812 | 0.641 | 0.542 | 28.1 |
| 103 | 1.147 | 0.807 | 0.646 | 0.539 | 28 |
| 104 | 1.172 | 0.819 | 0.649 | 0.55 | 28 |
| 105 | 1.195 | 0.838 | 0.664 | 0.566 | 28 |
| 106 | 1.236 | 0.856 | 0.671 | 0.578 | 28 |
| 107 | 1.25 | 0.872 | 0.667 | 0.576 | 28.1 |
| 108 | 1.225 | 0.843 | 0.654 | 0.567 | 28 |
| 109 | 1.204 | 0.816 | 0.641 | 0.554 | 28.1 |
| 110 | 1.174 | 0.808 | 0.633 | 0.542 | 28 |
| 111 | 1.148 | 0.806 | 0.628 | 0.543 | 28 |
| 112 | 1.144 | 0.825 | 0.65 | 0.548 | 28 |
| 113 | 1.171 | 0.839 | 0.656 | 0.56 | 28 |
| 114 | 1.201 | 0.867 | 0.668 | 0.578 | 28.1 |
| 115 | 1.234 | 0.85 | 0.674 | 0.586 | 28.1 |
| 116 | 1.232 | 0.829 | 0.662 | 0.584 | 28.1 |
| 117 | 1.201 | 0.803 | 0.649 | 0.566 | 28 |
| 118 | 1.144 | 0.791 | 0.634 | 0.557 | 28.1 |
| 119 | 1.129 | 0.806 | 0.623 | 0.547 | 28.1 |
| 120 | 1.142 | 0.817 | 0.63 | 0.558 | 28.1 |
| 121 | 1.208 | 0.847 | 0.65 | 0.573 | 28.1 |
| 122 | 1.245 | 0.849 | 0.673 | 0.581 | 28.1 |
| 123 | 1.248 | 0.84 | 0.679 | 0.594 | 28.1 |
| 124 | 1.216 | 0.82 | 0.654 | 0.596 | 28.1 |
| 125 | 1.171 | 0.808 | 0.645 | 0.579 | 28.1 |
| 126 | 1.115 | 0.793 | 0.635 | 0.567 | 28.1 |
| 127 | 1.108 | 0.804 | 0.632 | 0.555 | 28.1 |
| 128 | 1.134 | 0.821 | 0.658 | 0.551 | 28.1 |
| 129 | 1.153 | 0.84 | 0.667 | 0.561 | 28.1 |
| 130 | 1.194 | 0.827 | 0.661 | 0.57 | 28.2 |
| 131 | 1.185 | 0.823 | 0.678 | 0.6 | 28.3 |
| 132 | 1.187 | 0.809 | 0.674 | 0.597 | 28.2 |
| 133 | 1.147 | 0.788 | 0.671 | 0.603 | 28.2 |
| 134 | 1.119 | 0.784 | 0.657 | 0.588 | 28.3 |
| 135 | 1.105 | 0.792 | 0.637 | 0.579 | 28.3 |
| 136 | 1.141 | 0.788 | 0.629 | 0.564 | 28.3 |
| 137 | 1.165 | 0.81 | 0.627 | 0.563 | 28.3 |
| 138 | 1.18 | 0.849 | 0.646 | 0.567 | 28.4 |
| 139 | 1.184 | 0.829 | 0.659 | 0.586 | 28.4 |
| 140 | 1.188 | 0.825 | 0.669 | 0.605 | 28.4 |
| 141 | 1.158 | 0.8 | 0.672 | 0.602 | 28.4 |
| 142 | 1.141 | 0.782 | 0.676 | 0.605 | 28.4 |
| 143 | 1.103 | 0.791 | 0.66 | 0.589 | 28.4 |
| 144 | 1.107 | 0.8 | 0.65 | 0.579 | 28.4 |
| 145 | 1.145 | 0.82 | 0.638 | 0.562 | 28.5 |
| 146 | 1.194 | 0.845 | 0.631 | 0.571 | 28.5 |
| 147 | 1.204 | 0.814 | 0.646 | 0.591 | 28.5 |
| 148 | 1.203 | 0.81 | 0.659 | 0.608 | 28.5 |
| 149 | 1.159 | 0.788 | 0.67 | 0.606 | 28.5 |
| 150 | 1.15 | 0.786 | 0.689 | 0.595 | 28.5 |
| 151 | 1.149 | 0.788 | 0.682 | 0.576 | 28.4 |
| 152 | 1.158 | 0.822 | 0.67 | 0.571 | 28.4 |
| 153 | 1.187 | 0.829 | 0.65 | 0.595 | 28.4 |
| 154 | 1.218 | 0.83 | 0.643 | 0.61 | 28.4 |
| 155 | 1.209 | 0.824 | 0.634 | 0.613 | 28.4 |
| 156 | 1.227 | 0.793 | 0.643 | 0.604 | 28.4 |
| 157 | 1.208 | 0.788 | 0.641 | 0.588 | 28.4 |
| 158 | 1.184 | 0.771 | 0.668 | 0.579 | 28.4 |
| 159 | 1.152 | 0.807 | 0.678 | 0.584 | 28.4 |
| 160 | 1.139 | 0.818 | 0.68 | 0.606 | 28.4 |
| 161 | 1.168 | 0.832 | 0.672 | 0.623 | 28.5 |
| 162 | 1.186 | 0.838 | 0.651 | 0.626 | 28.6 |
| 163 | 1.197 | 0.817 | 0.644 | 0.601 | 28.6 |
| 164 | 1.217 | 0.799 | 0.654 | 0.583 | 28.6 |
| 165 | 1.207 | 0.798 | 0.658 | 0.578 | 28.6 |
| 166 | 1.202 | 0.781 | 0.673 | 0.586 | 28.7 |
| 167 | 1.16 | 0.771 | 0.694 | 0.617 | 28.6 |
| 168 | 1.113 | 0.782 | 0.69 | 0.63 | 28.6 |
| 169 | 1.125 | 0.792 | 0.688 | 0.616 | 28.7 |
| 170 | 1.147 | 0.808 | 0.666 | 0.595 | 28.7 |
| 171 | 1.189 | 0.819 | 0.655 | 0.58 | 28.7 |
| 172 | 1.208 | 0.821 | 0.652 | 0.589 | 28.7 |
| 173 | 1.193 | 0.837 | 0.642 | 0.613 | 28.7 |
| 174 | 1.15 | 0.807 | 0.662 | 0.632 | 28.7 |
| 175 | 1.125 | 0.796 | 0.687 | 0.629 | 28.7 |
| 176 | 1.128 | 0.788 | 0.691 | 0.614 | 28.7 |
| 177 | 1.175 | 0.786 | 0.697 | 0.596 | 28.7 |
| 178 | 1.177 | 0.798 | 0.668 | 0.588 | 28.7 |
| 179 | 1.159 | 0.846 | 0.651 | 0.606 | 28.7 |
| 180 | 1.119 | 0.858 | 0.649 | 0.613 | 28.7 |
| 181 | 1.136 | 0.824 | 0.651 | 0.619 | 28.6 |
| 182 | 1.176 | 0.812 | 0.663 | 0.64 | 28.6 |
| 183 | 1.199 | 0.8 | 0.693 | 0.632 | 28.6 |
| 184 | 1.185 | 0.789 | 0.682 | 0.624 | 28.6 |
| 185 | 1.159 | 0.792 | 0.698 | 0.605 | 28.6 |
| 186 | 1.122 | 0.807 | 0.693 | 0.597 | 28.6 |
| 187 | 1.116 | 0.824 | 0.675 | 0.597 | 28.6 |
| 188 | 1.142 | 0.831 | 0.658 | 0.615 | 28.6 |
| 189 | 1.164 | 0.83 | 0.648 | 0.624 | 28.5 |
| 190 | 1.177 | 0.822 | 0.648 | 0.645 | 28.6 |
| 191 | 1.192 | 0.794 | 0.648 | 0.634 | 28.6 |
| 192 | 1.156 | 0.79 | 0.674 | 0.621 | 28.6 |
| 193 | 1.152 | 0.78 | 0.695 | 0.598 | 28.7 |
| 194 | 1.098 | 0.774 | 0.687 | 0.605 | 28.7 |
| 195 | 1.131 | 0.814 | 0.689 | 0.602 | 28.8 |
| 196 | 1.167 | 0.819 | 0.693 | 0.616 | 28.8 |
| 197 | 1.185 | 0.821 | 0.677 | 0.638 | 28.8 |
| 198 | 1.201 | 0.827 | 0.652 | 0.648 | 28.9 |
| 199 | 1.153 | 0.799 | 0.671 | 0.649 | 28.9 |
| 200 | 1.115 | 0.79 | 0.651 | 0.636 | 28.9 |

Torque (Nm)

Temperature (°C)

|  | Wheat | Te10% | Te15% | Te20% | Dough temperature (°C) |
| --- | --- | --- | --- | --- | --- |
| 1 | 0 | 0 | 0.004 | 0 | 26.2 |
| 2 | 0.001 | 0 | 0 | 0.004 | 26.2 |
| 3 | 0.022 | 0.034 | 0.016 | 0.023 | 26.2 |
| 4 | 0.072 | 0.077 | 0.048 | 0.046 | 26.2 |
| 5 | 0.101 | 0.096 | 0.063 | 0.062 | 26.2 |
| 6 | 0.143 | 0.11 | 0.089 | 0.09 | 26.2 |
| 7 | 0.166 | 0.129 | 0.111 | 0.109 | 26.2 |
| 8 | 0.212 | 0.168 | 0.144 | 0.134 | 26.1 |
| 9 | 0.318 | 0.196 | 0.153 | 0.172 | 26.2 |
| 10 | 0.33 | 0.215 | 0.156 | 0.196 | 26.3 |
| 11 | 0.411 | 0.265 | 0.201 | 0.213 | 26.3 |
| 12 | 0.5 | 0.343 | 0.24 | 0.237 | 26.3 |
| 13 | 0.645 | 0.397 | 0.296 | 0.288 | 26.3 |
| 14 | 0.671 | 0.464 | 0.298 | 0.359 | 26.3 |
| 15 | 0.687 | 0.505 | 0.323 | 0.384 | 26.4 |
| 16 | 0.762 | 0.576 | 0.365 | 0.43 | 26.4 |
| 17 | 0.822 | 0.627 | 0.402 | 0.456 | 26.4 |
| 18 | 0.799 | 0.692 | 0.409 | 0.49 | 26.4 |
| 19 | 0.804 | 0.751 | 0.396 | 0.515 | 26.5 |
| 20 | 0.777 | 0.789 | 0.428 | 0.515 | 26.5 |
| 21 | 0.771 | 0.798 | 0.441 | 0.522 | 26.5 |
| 22 | 0.794 | 0.733 | 0.43 | 0.576 | 26.6 |
| 23 | 0.858 | 0.743 | 0.431 | 0.574 | 26.6 |
| 24 | 0.905 | 0.786 | 0.453 | 0.547 | 26.6 |
| 25 | 0.938 | 0.78 | 0.444 | 0.547 | 26.7 |
| 26 | 0.894 | 0.798 | 0.468 | 0.562 | 26.8 |
| 27 | 0.883 | 0.832 | 0.494 | 0.59 | 26.8 |
| 28 | 0.946 | 0.819 | 0.513 | 0.621 | 26.8 |
| 29 | 0.99 | 0.85 | 0.516 | 0.619 | 26.9 |
| 30 | 0.977 | 0.897 | 0.505 | 0.608 | 26.9 |
| 31 | 0.96 | 0.929 | 0.523 | 0.61 | 26.9 |
| 32 | 0.964 | 0.921 | 0.535 | 0.591 | 26.9 |
| 33 | 0.972 | 0.859 | 0.516 | 0.583 | 26.9 |
| 34 | 0.985 | 0.82 | 0.552 | 0.61 | 26.9 |
| 35 | 0.986 | 0.847 | 0.538 | 0.602 | 26.9 |
| 36 | 1.001 | 0.891 | 0.515 | 0.617 | 26.9 |
| 37 | 1.005 | 0.908 | 0.52 | 0.611 | 27 |
| 38 | 0.997 | 0.916 | 0.524 | 0.594 | 27 |
| 39 | 0.999 | 0.885 | 0.535 | 0.593 | 27 |
| 40 | 1.022 | 0.867 | 0.536 | 0.613 | 27 |
| 41 | 1.057 | 0.888 | 0.55 | 0.604 | 27.1 |
| 42 | 1.054 | 0.927 | 0.537 | 0.63 | 27.1 |
| 43 | 1.045 | 0.923 | 0.536 | 0.624 | 27.1 |
| 44 | 1.032 | 0.93 | 0.521 | 0.61 | 27.2 |
| 45 | 1.082 | 0.912 | 0.532 | 0.6 | 27.2 |
| 46 | 1.101 | 0.886 | 0.54 | 0.625 | 27.3 |
| 47 | 1.131 | 0.877 | 0.551 | 0.643 | 27.3 |
| 48 | 1.098 | 0.896 | 0.562 | 0.634 | 27.3 |
| 49 | 1.065 | 0.9 | 0.55 | 0.615 | 27.4 |
| 50 | 1.084 | 0.917 | 0.55 | 0.622 | 27.4 |
| 51 | 1.106 | 0.954 | 0.541 | 0.61 | 27.4 |
| 52 | 1.134 | 0.93 | 0.533 | 0.633 | 27.4 |
| 53 | 1.196 | 0.915 | 0.55 | 0.645 | 27.4 |
| 54 | 1.192 | 0.897 | 0.56 | 0.652 | 27.4 |
| 55 | 1.138 | 0.861 | 0.569 | 0.621 | 27.4 |
| 56 | 1.117 | 0.896 | 0.557 | 0.616 | 27.4 |
| 57 | 1.143 | 0.918 | 0.541 | 0.625 | 27.4 |
| 58 | 1.177 | 0.954 | 0.544 | 0.645 | 27.5 |
| 59 | 1.199 | 0.956 | 0.562 | 0.657 | 27.5 |
| 60 | 1.173 | 0.926 | 0.571 | 0.64 | 27.5 |
| 61 | 1.145 | 0.888 | 0.581 | 0.62 | 27.5 |
| 62 | 1.116 | 0.871 | 0.577 | 0.611 | 27.4 |
| 63 | 1.113 | 0.873 | 0.56 | 0.63 | 27.5 |
| 64 | 1.135 | 0.917 | 0.557 | 0.656 | 27.5 |
| 65 | 1.183 | 0.953 | 0.539 | 0.661 | 27.6 |
| 66 | 1.209 | 0.949 | 0.548 | 0.636 | 27.6 |
| 67 | 1.204 | 0.902 | 0.562 | 0.63 | 27.6 |
| 68 | 1.174 | 0.875 | 0.568 | 0.62 | 27.5 |
| 69 | 1.142 | 0.886 | 0.582 | 0.615 | 27.5 |
| 70 | 1.127 | 0.932 | 0.579 | 0.626 | 27.5 |
| 71 | 1.176 | 0.959 | 0.569 | 0.644 | 27.5 |
| 72 | 1.218 | 0.969 | 0.561 | 0.647 | 27.6 |
| 73 | 1.23 | 0.92 | 0.545 | 0.661 | 27.6 |
| 74 | 1.208 | 0.887 | 0.542 | 0.648 | 27.6 |
| 75 | 1.16 | 0.878 | 0.538 | 0.626 | 27.6 |
| 76 | 1.152 | 0.889 | 0.543 | 0.631 | 27.6 |
| 77 | 1.187 | 0.921 | 0.565 | 0.624 | 27.6 |
| 78 | 1.222 | 0.938 | 0.58 | 0.615 | 27.6 |
| 79 | 1.239 | 0.945 | 0.56 | 0.64 | 27.6 |
| 80 | 1.244 | 0.953 | 0.566 | 0.644 | 27.6 |
| 81 | 1.195 | 0.926 | 0.557 | 0.657 | 27.6 |
| 82 | 1.17 | 0.894 | 0.536 | 0.659 | 27.6 |
| 83 | 1.156 | 0.869 | 0.543 | 0.649 | 27.6 |
| 84 | 1.209 | 0.879 | 0.557 | 0.629 | 27.6 |
| 85 | 1.245 | 0.915 | 0.57 | 0.623 | 27.6 |
| 86 | 1.241 | 0.92 | 0.577 | 0.624 | 27.7 |
| 87 | 1.252 | 0.944 | 0.574 | 0.633 | 27.7 |
| 88 | 1.193 | 0.941 | 0.564 | 0.642 | 27.8 |
| 89 | 1.168 | 0.93 | 0.541 | 0.653 | 27.8 |
| 90 | 1.177 | 0.913 | 0.539 | 0.66 | 27.8 |
| 91 | 1.228 | 0.878 | 0.541 | 0.644 | 27.8 |
| 92 | 1.253 | 0.879 | 0.546 | 0.646 | 27.9 |
| 93 | 1.257 | 0.887 | 0.564 | 0.627 | 27.9 |
| 94 | 1.214 | 0.926 | 0.58 | 0.615 | 28 |
| 95 | 1.187 | 0.951 | 0.571 | 0.626 | 28 |
| 96 | 1.174 | 0.953 | 0.567 | 0.636 | 27.9 |
| 97 | 1.209 | 0.916 | 0.562 | 0.642 | 28 |
| 98 | 1.246 | 0.874 | 0.537 | 0.664 | 28 |
| 99 | 1.27 | 0.877 | 0.527 | 0.664 | 28 |
| 100 | 1.256 | 0.882 | 0.536 | 0.654 | 28.1 |
| 101 | 1.223 | 0.909 | 0.538 | 0.64 | 28.1 |
| 102 | 1.171 | 0.918 | 0.554 | 0.63 | 28.1 |
| 103 | 1.147 | 0.939 | 0.567 | 0.62 | 28 |
| 104 | 1.172 | 0.933 | 0.565 | 0.624 | 28 |
| 105 | 1.195 | 0.939 | 0.573 | 0.631 | 28 |
| 106 | 1.236 | 0.911 | 0.56 | 0.642 | 28 |
| 107 | 1.25 | 0.886 | 0.542 | 0.65 | 28.1 |
| 108 | 1.225 | 0.856 | 0.533 | 0.655 | 28 |
| 109 | 1.204 | 0.87 | 0.53 | 0.659 | 28.1 |
| 110 | 1.174 | 0.892 | 0.54 | 0.647 | 28 |
| 111 | 1.148 | 0.94 | 0.554 | 0.63 | 28 |
| 112 | 1.144 | 0.935 | 0.561 | 0.623 | 28 |
| 113 | 1.171 | 0.911 | 0.57 | 0.619 | 28 |
| 114 | 1.201 | 0.884 | 0.569 | 0.634 | 28.1 |
| 115 | 1.234 | 0.872 | 0.549 | 0.654 | 28.1 |
| 116 | 1.232 | 0.881 | 0.532 | 0.656 | 28.1 |
| 117 | 1.201 | 0.898 | 0.518 | 0.654 | 28 |
| 118 | 1.144 | 0.942 | 0.531 | 0.64 | 28.1 |
| 119 | 1.129 | 0.945 | 0.551 | 0.626 | 28.1 |
| 120 | 1.142 | 0.931 | 0.563 | 0.615 | 28.1 |
| 121 | 1.208 | 0.909 | 0.569 | 0.618 | 28.1 |
| 122 | 1.245 | 0.877 | 0.566 | 0.629 | 28.1 |
| 123 | 1.248 | 0.858 | 0.553 | 0.649 | 28.1 |
| 124 | 1.216 | 0.876 | 0.542 | 0.657 | 28.1 |
| 125 | 1.171 | 0.897 | 0.529 | 0.664 | 28.1 |
| 126 | 1.115 | 0.906 | 0.523 | 0.65 | 28.1 |
| 127 | 1.108 | 0.91 | 0.53 | 0.634 | 28.1 |
| 128 | 1.134 | 0.917 | 0.546 | 0.622 | 28.1 |
| 129 | 1.153 | 0.919 | 0.562 | 0.613 | 28.1 |
| 130 | 1.194 | 0.892 | 0.562 | 0.637 | 28.2 |
| 131 | 1.185 | 0.863 | 0.562 | 0.659 | 28.3 |
| 132 | 1.187 | 0.85 | 0.551 | 0.666 | 28.2 |
| 133 | 1.147 | 0.879 | 0.533 | 0.656 | 28.2 |
| 134 | 1.119 | 0.881 | 0.521 | 0.634 | 28.3 |
| 135 | 1.105 | 0.921 | 0.529 | 0.618 | 28.3 |
| 136 | 1.141 | 0.929 | 0.544 | 0.614 | 28.3 |
| 137 | 1.165 | 0.922 | 0.557 | 0.633 | 28.3 |
| 138 | 1.18 | 0.916 | 0.57 | 0.66 | 28.4 |
| 139 | 1.184 | 0.876 | 0.567 | 0.667 | 28.4 |
| 140 | 1.188 | 0.848 | 0.562 | 0.664 | 28.4 |
| 141 | 1.158 | 0.857 | 0.544 | 0.659 | 28.4 |
| 142 | 1.141 | 0.856 | 0.525 | 0.634 | 28.4 |
| 143 | 1.103 | 0.884 | 0.522 | 0.624 | 28.4 |
| 144 | 1.107 | 0.914 | 0.529 | 0.631 | 28.4 |
| 145 | 1.145 | 0.908 | 0.544 | 0.628 | 28.5 |
| 146 | 1.194 | 0.887 | 0.558 | 0.639 | 28.5 |
| 147 | 1.204 | 0.851 | 0.571 | 0.667 | 28.5 |
| 148 | 1.203 | 0.854 | 0.567 | 0.67 | 28.5 |
| 149 | 1.159 | 0.858 | 0.553 | 0.661 | 28.5 |
| 150 | 1.15 | 0.893 | 0.533 | 0.639 | 28.5 |
| 151 | 1.149 | 0.903 | 0.526 | 0.614 | 28.4 |
| 152 | 1.158 | 0.901 | 0.525 | 0.618 | 28.4 |
| 153 | 1.187 | 0.909 | 0.529 | 0.627 | 28.4 |
| 154 | 1.218 | 0.881 | 0.545 | 0.645 | 28.4 |
| 155 | 1.209 | 0.864 | 0.568 | 0.667 | 28.4 |
| 156 | 1.227 | 0.836 | 0.572 | 0.67 | 28.4 |
| 157 | 1.208 | 0.848 | 0.568 | 0.671 | 28.4 |
| 158 | 1.184 | 0.861 | 0.541 | 0.667 | 28.4 |
| 159 | 1.152 | 0.876 | 0.522 | 0.647 | 28.4 |
| 160 | 1.139 | 0.898 | 0.523 | 0.627 | 28.4 |
| 161 | 1.168 | 0.922 | 0.526 | 0.622 | 28.5 |
| 162 | 1.186 | 0.899 | 0.535 | 0.621 | 28.6 |
| 163 | 1.197 | 0.858 | 0.56 | 0.651 | 28.6 |
| 164 | 1.217 | 0.832 | 0.576 | 0.658 | 28.6 |
| 165 | 1.207 | 0.848 | 0.556 | 0.67 | 28.6 |
| 166 | 1.202 | 0.871 | 0.544 | 0.667 | 28.7 |
| 167 | 1.16 | 0.892 | 0.537 | 0.637 | 28.6 |
| 168 | 1.113 | 0.903 | 0.526 | 0.625 | 28.6 |
| 169 | 1.125 | 0.909 | 0.525 | 0.627 | 28.7 |
| 170 | 1.147 | 0.891 | 0.545 | 0.643 | 28.7 |
| 171 | 1.189 | 0.891 | 0.558 | 0.661 | 28.7 |
| 172 | 1.208 | 0.842 | 0.569 | 0.676 | 28.7 |
| 173 | 1.193 | 0.835 | 0.567 | 0.666 | 28.7 |
| 174 | 1.15 | 0.847 | 0.549 | 0.663 | 28.7 |
| 175 | 1.125 | 0.858 | 0.546 | 0.626 | 28.7 |
| 176 | 1.128 | 0.896 | 0.527 | 0.625 | 28.7 |
| 177 | 1.175 | 0.893 | 0.521 | 0.623 | 28.7 |
| 178 | 1.177 | 0.865 | 0.529 | 0.645 | 28.7 |
| 179 | 1.159 | 0.843 | 0.533 | 0.674 | 28.7 |
| 180 | 1.119 | 0.816 | 0.546 | 0.676 | 28.7 |
| 181 | 1.136 | 0.844 | 0.562 | 0.672 | 28.6 |
| 182 | 1.176 | 0.853 | 0.571 | 0.651 | 28.6 |
| 183 | 1.199 | 0.874 | 0.564 | 0.63 | 28.6 |
| 184 | 1.185 | 0.898 | 0.549 | 0.619 | 28.6 |
| 185 | 1.159 | 0.892 | 0.531 | 0.642 | 28.6 |
| 186 | 1.122 | 0.861 | 0.527 | 0.654 | 28.6 |
| 187 | 1.116 | 0.851 | 0.534 | 0.666 | 28.6 |
| 188 | 1.142 | 0.815 | 0.556 | 0.676 | 28.6 |
| 189 | 1.164 | 0.826 | 0.564 | 0.67 | 28.5 |
| 190 | 1.177 | 0.865 | 0.581 | 0.654 | 28.6 |
| 191 | 1.192 | 0.882 | 0.563 | 0.635 | 28.6 |
| 192 | 1.156 | 0.883 | 0.553 | 0.621 | 28.6 |
| 193 | 1.152 | 0.908 | 0.54 | 0.63 | 28.7 |
| 194 | 1.098 | 0.874 | 0.533 | 0.64 | 28.7 |
| 195 | 1.131 | 0.846 | 0.534 | 0.669 | 28.8 |
| 196 | 1.167 | 0.825 | 0.53 | 0.677 | 28.8 |
| 197 | 1.185 | 0.81 | 0.549 | 0.677 | 28.8 |
| 198 | 1.201 | 0.82 | 0.569 | 0.669 | 28.9 |
| 199 | 1.153 | 0.837 | 0.576 | 0.644 | 28.9 |
| 200 | 1.115 | 0.881 | 0.567 | 0.618 | 28.9 |

Torque (Nm)

Temperature (°C)

|  | Wheat | S10 | S15 | S20% | Block temperature (°C) | Dough temperature (°C) | Wheat | S10 | S15 | S20% | Dough temperature (°C) |
| --- | --- | --- | --- | --- | --- | --- | --- | --- | --- | --- | --- |
| 1 | 0 | 0 | 0 | 0 | 30.4 | 26.2 | 0 | 0 | 0 | 0 | 26.2 |
| 2 | 0.001 | 0 | 0.001 | 0 | 30.4 | 26.2 | 0.001 | 0 | 0.001 | 0 | 26.2 |
| 3 | 0.022 | 0.018 | 0.014 | 0.024 | 30.4 | 26.2 | 0.022 | 0.018 | 0.014 | 0.024 | 26.2 |
| 4 | 0.072 | 0.068 | 0.056 | 0.079 | 30.4 | 26.2 | 0.072 | 0.068 | 0.056 | 0.079 | 26.2 |
| 5 | 0.101 | 0.099 | 0.075 | 0.117 | 30.5 | 26.2 | 0.101 | 0.099 | 0.075 | 0.117 | 26.2 |
| 6 | 0.143 | 0.129 | 0.099 | 0.126 | 30.5 | 26.2 | 0.143 | 0.129 | 0.099 | 0.126 | 26.2 |
| 7 | 0.166 | 0.17 | 0.133 | 0.15 | 30.5 | 26.2 | 0.166 | 0.17 | 0.133 | 0.15 | 26.2 |
| 8 | 0.212 | 0.23 | 0.154 | 0.178 | 30.5 | 26.1 | 0.212 | 0.23 | 0.154 | 0.178 | 26.1 |
| 9 | 0.318 | 0.285 | 0.186 | 0.223 | 30.5 | 26.2 | 0.318 | 0.285 | 0.186 | 0.223 | 26.2 |
| 10 | 0.33 | 0.35 | 0.221 | 0.238 | 30.5 | 26.3 | 0.33 | 0.35 | 0.221 | 0.238 | 26.3 |
| 11 | 0.411 | 0.42 | 0.223 | 0.293 | 30.5 | 26.3 | 0.411 | 0.42 | 0.223 | 0.293 | 26.3 |
| 12 | 0.5 | 0.546 | 0.322 | 0.412 | 30.6 | 26.3 | 0.5 | 0.546 | 0.322 | 0.412 | 26.3 |
| 13 | 0.645 | 0.603 | 0.408 | 0.47 | 30.6 | 26.3 | 0.645 | 0.603 | 0.408 | 0.47 | 26.3 |
| 14 | 0.671 | 0.65 | 0.458 | 0.497 | 30.6 | 26.3 | 0.671 | 0.65 | 0.458 | 0.497 | 26.3 |
| 15 | 0.687 | 0.714 | 0.486 | 0.539 | 30.6 | 26.4 | 0.687 | 0.714 | 0.486 | 0.539 | 26.4 |
| 16 | 0.762 | 0.754 | 0.531 | 0.647 | 30.6 | 26.4 | 0.762 | 0.754 | 0.531 | 0.647 | 26.4 |
| 17 | 0.822 | 0.755 | 0.622 | 0.712 | 30.6 | 26.4 | 0.822 | 0.755 | 0.622 | 0.712 | 26.4 |
| 18 | 0.799 | 0.723 | 0.658 | 0.802 | 30.6 | 26.4 | 0.799 | 0.723 | 0.658 | 0.802 | 26.4 |
| 19 | 0.804 | 0.729 | 0.792 | 0.899 | 30.7 | 26.5 | 0.804 | 0.729 | 0.792 | 0.899 | 26.5 |
| 20 | 0.777 | 0.757 | 0.843 | 0.903 | 30.7 | 26.5 | 0.777 | 0.757 | 0.843 | 0.903 | 26.5 |
| 21 | 0.771 | 0.814 | 0.87 | 0.903 | 30.7 | 26.5 | 0.771 | 0.814 | 0.87 | 0.903 | 26.5 |
| 22 | 0.794 | 0.83 | 0.929 | 0.942 | 30.7 | 26.6 | 0.794 | 0.83 | 0.929 | 0.942 | 26.6 |
| 23 | 0.858 | 0.835 | 0.955 | 1.023 | 30.7 | 26.6 | 0.858 | 0.835 | 0.955 | 1.023 | 26.6 |
| 24 | 0.905 | 0.836 | 0.973 | 1.065 | 30.7 | 26.6 | 0.905 | 0.836 | 0.973 | 1.065 | 26.6 |
| 25 | 0.938 | 0.88 | 0.983 | 1.081 | 30.7 | 26.7 | 0.938 | 0.88 | 0.983 | 1.081 | 26.7 |
| 26 | 0.894 | 0.917 | 0.944 | 1.053 | 30.7 | 26.8 | 0.894 | 0.917 | 0.944 | 1.053 | 26.8 |
| 27 | 0.883 | 0.948 | 0.927 | 1.098 | 30.7 | 26.8 | 0.883 | 0.948 | 0.927 | 1.098 | 26.8 |
| 28 | 0.946 | 0.911 | 0.929 | 1.244 | 30.7 | 26.8 | 0.946 | 0.911 | 0.929 | 1.244 | 26.8 |
| 29 | 0.99 | 0.897 | 0.986 | 1.308 | 30.7 | 26.9 | 0.99 | 0.897 | 0.986 | 1.308 | 26.9 |
| 30 | 0.977 | 0.93 | 1.026 | 1.302 | 30.7 | 26.9 | 0.977 | 0.93 | 1.026 | 1.302 | 26.9 |
| 31 | 0.96 | 0.956 | 1.003 | 1.298 | 30.8 | 26.9 | 0.96 | 0.956 | 1.003 | 1.298 | 26.9 |
| 32 | 0.964 | 0.9 | 0.961 | 1.414 | 30.8 | 26.9 | 0.964 | 0.9 | 0.961 | 1.414 | 26.9 |
| 33 | 0.972 | 0.8 | 0.887 | 1.503 | 30.8 | 26.9 | 0.972 | 0.8 | 0.887 | 1.503 | 26.9 |
| 34 | 0.985 | 0.748 | 0.74 | 1.462 | 30.8 | 26.9 | 0.985 | 0.748 | 0.74 | 1.462 | 26.9 |
| 35 | 0.986 | 0.751 | 0.7 | 1.384 | 30.8 | 26.9 | 0.986 | 0.751 | 0.7 | 1.384 | 26.9 |
| 36 | 1.001 | 0.771 | 0.707 | 1.334 | 30.8 | 26.9 | 1.001 | 0.771 | 0.707 | 1.334 | 26.9 |
| 37 | 1.005 | 0.758 | 0.699 | 1.5 | 30.8 | 27 | 1.005 | 0.758 | 0.699 | 1.5 | 27 |
| 38 | 0.997 | 0.771 | 0.725 | 1.619 | 30.8 | 27 | 0.997 | 0.771 | 0.725 | 1.619 | 27 |
| 39 | 0.999 | 0.802 | 0.741 | 1.599 | 30.8 | 27 | 0.999 | 0.802 | 0.741 | 1.599 | 27 |
| 40 | 1.022 | 0.854 | 0.772 | 1.543 | 30.8 | 27 | 1.022 | 0.854 | 0.772 | 1.543 | 27 |
| 41 | 1.057 | 0.875 | 0.79 | 1.536 | 30.8 | 27.1 | 1.057 | 0.875 | 0.79 | 1.536 | 27.1 |
| 42 | 1.054 | 0.878 | 0.818 | 1.603 | 30.8 | 27.1 | 1.054 | 0.878 | 0.818 | 1.603 | 27.1 |
| 43 | 1.045 | 0.848 | 0.909 | 1.725 | 30.8 | 27.1 | 1.045 | 0.848 | 0.909 | 1.725 | 27.1 |
| 44 | 1.032 | 0.86 | 0.936 | 1.756 | 30.8 | 27.2 | 1.032 | 0.86 | 0.936 | 1.756 | 27.2 |
| 45 | 1.082 | 0.911 | 0.946 | 1.705 | 30.8 | 27.2 | 1.082 | 0.911 | 0.946 | 1.705 | 27.2 |
| 46 | 1.101 | 0.916 | 0.97 | 1.644 | 30.8 | 27.3 | 1.101 | 0.916 | 0.97 | 1.644 | 27.3 |
| 47 | 1.131 | 0.912 | 0.969 | 1.59 | 30.8 | 27.3 | 1.131 | 0.912 | 0.969 | 1.59 | 27.3 |
| 48 | 1.098 | 0.901 | 1.009 | 1.673 | 30.8 | 27.3 | 1.098 | 0.901 | 1.009 | 1.673 | 27.3 |
| 49 | 1.065 | 0.886 | 1.018 | 1.771 | 30.8 | 27.4 | 1.065 | 0.886 | 1.018 | 1.771 | 27.4 |
| 50 | 1.084 | 0.883 | 0.99 | 1.883 | 30.8 | 27.4 | 1.084 | 0.883 | 0.99 | 1.883 | 27.4 |
| 51 | 1.106 | 0.927 | 0.998 | 1.814 | 30.8 | 27.4 | 1.106 | 0.927 | 0.998 | 1.814 | 27.4 |
| 52 | 1.134 | 0.958 | 1.067 | 1.74 | 30.8 | 27.4 | 1.134 | 0.958 | 1.067 | 1.74 | 27.4 |
| 53 | 1.196 | 0.954 | 1.106 | 1.695 | 30.8 | 27.4 | 1.196 | 0.954 | 1.106 | 1.695 | 27.4 |
| 54 | 1.192 | 0.949 | 1.109 | 1.67 | 30.8 | 27.4 | 1.192 | 0.949 | 1.109 | 1.67 | 27.4 |
| 55 | 1.138 | 0.913 | 1.118 | 1.813 | 30.8 | 27.4 | 1.138 | 0.913 | 1.118 | 1.813 | 27.4 |
| 56 | 1.117 | 0.913 | 1.106 | 1.9 | 30.8 | 27.4 | 1.117 | 0.913 | 1.106 | 1.9 | 27.4 |
| 57 | 1.143 | 0.923 | 1.069 | 1.899 | 30.8 | 27.4 | 1.143 | 0.923 | 1.069 | 1.899 | 27.4 |
| 58 | 1.177 | 0.962 | 1.153 | 1.795 | 30.8 | 27.5 | 1.177 | 0.962 | 1.153 | 1.795 | 27.5 |
| 59 | 1.199 | 0.999 | 1.185 | 1.703 | 30.8 | 27.5 | 1.199 | 0.999 | 1.185 | 1.703 | 27.5 |
| 60 | 1.173 | 0.965 | 1.204 | 1.712 | 30.8 | 27.5 | 1.173 | 0.965 | 1.204 | 1.712 | 27.5 |
| 61 | 1.145 | 0.938 | 1.174 | 1.742 | 30.8 | 27.5 | 1.145 | 0.938 | 1.174 | 1.742 | 27.5 |
| 62 | 1.116 | 0.94 | 1.152 | 1.892 | 30.8 | 27.4 | 1.116 | 0.94 | 1.152 | 1.892 | 27.4 |
| 63 | 1.113 | 0.913 | 1.171 | 1.916 | 30.7 | 27.5 | 1.113 | 0.913 | 1.171 | 1.916 | 27.5 |
| 64 | 1.135 | 0.923 | 1.194 | 1.883 | 30.7 | 27.5 | 1.135 | 0.923 | 1.194 | 1.883 | 27.5 |
| 65 | 1.183 | 0.96 | 1.257 | 1.895 | 30.7 | 27.6 | 1.183 | 0.96 | 1.257 | 1.895 | 27.6 |
| 66 | 1.209 | 0.969 | 1.258 | 1.749 | 30.7 | 27.6 | 1.209 | 0.969 | 1.258 | 1.749 | 27.6 |
| 67 | 1.204 | 0.985 | 1.199 | 1.752 | 30.7 | 27.6 | 1.204 | 0.985 | 1.199 | 1.752 | 27.6 |
| 68 | 1.174 | 0.963 | 1.213 | 1.809 | 30.7 | 27.5 | 1.174 | 0.963 | 1.213 | 1.809 | 27.5 |
| 69 | 1.142 | 0.932 | 1.156 | 1.923 | 30.7 | 27.5 | 1.142 | 0.932 | 1.156 | 1.923 | 27.5 |
| 70 | 1.127 | 0.937 | 1.145 | 2.004 | 30.7 | 27.5 | 1.127 | 0.937 | 1.145 | 2.004 | 27.5 |
| 71 | 1.176 | 0.951 | 1.188 | 1.912 | 30.7 | 27.5 | 1.176 | 0.951 | 1.188 | 1.912 | 27.5 |
| 72 | 1.218 | 0.98 | 1.21 | 1.841 | 30.7 | 27.6 | 1.218 | 0.98 | 1.21 | 1.841 | 27.6 |
| 73 | 1.23 | 0.975 | 1.241 | 1.795 | 30.7 | 27.6 | 1.23 | 0.975 | 1.241 | 1.795 | 27.6 |
| 74 | 1.208 | 0.988 | 1.224 | 1.729 | 30.7 | 27.6 | 1.208 | 0.988 | 1.224 | 1.729 | 27.6 |
| 75 | 1.16 | 0.981 | 1.203 | 1.859 | 30.7 | 27.6 | 1.16 | 0.981 | 1.203 | 1.859 | 27.6 |
| 76 | 1.152 | 0.933 | 1.18 | 1.92 | 30.7 | 27.6 | 1.152 | 0.933 | 1.18 | 1.92 | 27.6 |
| 77 | 1.187 | 0.921 | 1.16 | 1.975 | 30.7 | 27.6 | 1.187 | 0.921 | 1.16 | 1.975 | 27.6 |
| 78 | 1.222 | 0.937 | 1.157 | 1.957 | 30.7 | 27.6 | 1.222 | 0.937 | 1.157 | 1.957 | 27.6 |
| 79 | 1.239 | 0.983 | 1.233 | 1.935 | 30.7 | 27.6 | 1.239 | 0.983 | 1.233 | 1.935 | 27.6 |
| 80 | 1.244 | 0.994 | 1.267 | 1.774 | 30.7 | 27.6 | 1.244 | 0.994 | 1.267 | 1.774 | 27.6 |
| 81 | 1.195 | 1.001 | 1.255 | 1.791 | 30.7 | 27.6 | 1.195 | 1.001 | 1.255 | 1.791 | 27.6 |
| 82 | 1.17 | 0.973 | 1.246 | 1.804 | 30.7 | 27.6 | 1.17 | 0.973 | 1.246 | 1.804 | 27.6 |
| 83 | 1.156 | 0.946 | 1.174 | 1.868 | 30.7 | 27.6 | 1.156 | 0.946 | 1.174 | 1.868 | 27.6 |
| 84 | 1.209 | 0.917 | 1.179 | 1.944 | 30.7 | 27.6 | 1.209 | 0.917 | 1.179 | 1.944 | 27.6 |
| 85 | 1.245 | 0.935 | 1.193 | 1.912 | 30.7 | 27.6 | 1.245 | 0.935 | 1.193 | 1.912 | 27.6 |
| 86 | 1.241 | 0.966 | 1.242 | 1.832 | 30.7 | 27.7 | 1.241 | 0.966 | 1.242 | 1.832 | 27.7 |
| 87 | 1.252 | 0.985 | 1.274 | 1.826 | 30.7 | 27.7 | 1.252 | 0.985 | 1.274 | 1.826 | 27.7 |
| 88 | 1.193 | 0.984 | 1.261 | 1.728 | 30.7 | 27.8 | 1.193 | 0.984 | 1.261 | 1.728 | 27.8 |
| 89 | 1.168 | 0.977 | 1.214 | 1.756 | 30.7 | 27.8 | 1.168 | 0.977 | 1.214 | 1.756 | 27.8 |
| 90 | 1.177 | 0.925 | 1.188 | 1.871 | 30.7 | 27.8 | 1.177 | 0.925 | 1.188 | 1.871 | 27.8 |
| 91 | 1.228 | 0.93 | 1.164 | 1.987 | 30.7 | 27.8 | 1.228 | 0.93 | 1.164 | 1.987 | 27.8 |
| 92 | 1.253 | 0.958 | 1.2 | 1.927 | 30.7 | 27.9 | 1.253 | 0.958 | 1.2 | 1.927 | 27.9 |
| 93 | 1.257 | 0.977 | 1.231 | 1.893 | 30.7 | 27.9 | 1.257 | 0.977 | 1.231 | 1.893 | 27.9 |
| 94 | 1.214 | 0.999 | 1.259 | 1.83 | 30.7 | 28 | 1.214 | 0.999 | 1.259 | 1.83 | 28 |
| 95 | 1.187 | 0.973 | 1.301 | 1.74 | 30.7 | 28 | 1.187 | 0.973 | 1.301 | 1.74 | 28 |
| 96 | 1.174 | 0.932 | 1.299 | 1.828 | 30.7 | 27.9 | 1.174 | 0.932 | 1.299 | 1.828 | 27.9 |
| 97 | 1.209 | 0.919 | 1.256 | 1.876 | 30.7 | 28 | 1.209 | 0.919 | 1.256 | 1.876 | 28 |
| 98 | 1.246 | 0.932 | 1.229 | 1.953 | 30.7 | 28 | 1.246 | 0.932 | 1.229 | 1.953 | 28 |
| 99 | 1.27 | 0.975 | 1.19 | 1.965 | 30.7 | 28 | 1.27 | 0.975 | 1.19 | 1.965 | 28 |
| 100 | 1.256 | 0.97 | 1.196 | 1.862 | 30.7 | 28.1 | 1.256 | 0.97 | 1.196 | 1.862 | 28.1 |
| 101 | 1.223 | 0.972 | 1.237 | 1.84 | 30.6 | 28.1 | 1.223 | 0.972 | 1.237 | 1.84 | 28.1 |
| 102 | 1.171 | 0.956 | 1.27 | 1.767 | 30.6 | 28.1 | 1.171 | 0.956 | 1.27 | 1.767 | 28.1 |
| 103 | 1.147 | 0.908 | 1.281 | 1.769 | 30.6 | 28 | 1.147 | 0.908 | 1.281 | 1.769 | 28 |
| 104 | 1.172 | 0.907 | 1.244 | 1.804 | 30.6 | 28 | 1.172 | 0.907 | 1.244 | 1.804 | 28 |
| 105 | 1.195 | 0.954 | 1.208 | 1.913 | 30.6 | 28 | 1.195 | 0.954 | 1.208 | 1.913 | 28 |
| 106 | 1.236 | 0.961 | 1.19 | 1.912 | 30.6 | 28 | 1.236 | 0.961 | 1.19 | 1.912 | 28 |
| 107 | 1.25 | 0.975 | 1.2 | 1.889 | 30.6 | 28.1 | 1.25 | 0.975 | 1.2 | 1.889 | 28.1 |
| 108 | 1.225 | 0.999 | 1.213 | 1.832 | 30.6 | 28 | 1.225 | 0.999 | 1.213 | 1.832 | 28 |
| 109 | 1.204 | 0.948 | 1.296 | 1.758 | 30.6 | 28.1 | 1.204 | 0.948 | 1.296 | 1.758 | 28.1 |
| 110 | 1.174 | 0.929 | 1.288 | 1.733 | 30.6 | 28 | 1.174 | 0.929 | 1.288 | 1.733 | 28 |
| 111 | 1.148 | 0.913 | 1.282 | 1.831 | 30.6 | 28 | 1.148 | 0.913 | 1.282 | 1.831 | 28 |
| 112 | 1.144 | 0.956 | 1.265 | 1.911 | 30.6 | 28 | 1.144 | 0.956 | 1.265 | 1.911 | 28 |
| 113 | 1.171 | 0.979 | 1.228 | 1.911 | 30.6 | 28 | 1.171 | 0.979 | 1.228 | 1.911 | 28 |
| 114 | 1.201 | 0.951 | 1.205 | 1.928 | 30.6 | 28.1 | 1.201 | 0.951 | 1.205 | 1.928 | 28.1 |
| 115 | 1.234 | 0.958 | 1.196 | 1.835 | 30.6 | 28.1 | 1.234 | 0.958 | 1.196 | 1.835 | 28.1 |
| 116 | 1.232 | 0.922 | 1.228 | 1.772 | 30.6 | 28.1 | 1.232 | 0.922 | 1.228 | 1.772 | 28.1 |
| 117 | 1.201 | 0.884 | 1.248 | 1.763 | 30.6 | 28 | 1.201 | 0.884 | 1.248 | 1.763 | 28 |
| 118 | 1.144 | 0.942 | 1.275 | 1.831 | 30.6 | 28.1 | 1.144 | 0.942 | 1.275 | 1.831 | 28.1 |
| 119 | 1.129 | 0.979 | 1.27 | 1.91 | 30.6 | 28.1 | 1.129 | 0.979 | 1.27 | 1.91 | 28.1 |
| 120 | 1.142 | 0.977 | 1.257 | 1.949 | 30.5 | 28.1 | 1.142 | 0.977 | 1.257 | 1.949 | 28.1 |
| 121 | 1.208 | 0.992 | 1.21 | 1.888 | 30.5 | 28.1 | 1.208 | 0.992 | 1.21 | 1.888 | 28.1 |
| 122 | 1.245 | 0.95 | 1.19 | 1.754 | 30.5 | 28.1 | 1.245 | 0.95 | 1.19 | 1.754 | 28.1 |
| 123 | 1.248 | 0.917 | 1.211 | 1.778 | 30.5 | 28.1 | 1.248 | 0.917 | 1.211 | 1.778 | 28.1 |
| 124 | 1.216 | 0.911 | 1.235 | 1.863 | 30.5 | 28.1 | 1.216 | 0.911 | 1.235 | 1.863 | 28.1 |
| 125 | 1.171 | 0.927 | 1.275 | 1.925 | 30.5 | 28.1 | 1.171 | 0.927 | 1.275 | 1.925 | 28.1 |
| 126 | 1.115 | 0.964 | 1.312 | 1.907 | 30.5 | 28.1 | 1.115 | 0.964 | 1.312 | 1.907 | 28.1 |
| 127 | 1.108 | 0.959 | 1.299 | 1.861 | 30.5 | 28.1 | 1.108 | 0.959 | 1.299 | 1.861 | 28.1 |
| 128 | 1.134 | 0.958 | 1.326 | 1.801 | 30.5 | 28.1 | 1.134 | 0.958 | 1.326 | 1.801 | 28.1 |
| 129 | 1.153 | 0.927 | 1.265 | 1.749 | 30.5 | 28.1 | 1.153 | 0.927 | 1.265 | 1.749 | 28.1 |
| 130 | 1.194 | 0.913 | 1.233 | 1.848 | 30.5 | 28.2 | 1.194 | 0.913 | 1.233 | 1.848 | 28.2 |
| 131 | 1.185 | 0.92 | 1.23 | 1.881 | 30.5 | 28.3 | 1.185 | 0.92 | 1.23 | 1.881 | 28.3 |
| 132 | 1.187 | 0.961 | 1.233 | 1.948 | 30.5 | 28.2 | 1.187 | 0.961 | 1.233 | 1.948 | 28.2 |
| 133 | 1.147 | 0.969 | 1.235 | 1.876 | 30.5 | 28.2 | 1.147 | 0.969 | 1.235 | 1.876 | 28.2 |
| 134 | 1.119 | 0.976 | 1.241 | 1.8 | 30.5 | 28.3 | 1.119 | 0.976 | 1.241 | 1.8 | 28.3 |
| 135 | 1.105 | 0.962 | 1.278 | 1.768 | 30.5 | 28.3 | 1.105 | 0.962 | 1.278 | 1.768 | 28.3 |
| 136 | 1.141 | 0.916 | 1.298 | 1.799 | 30.5 | 28.3 | 1.141 | 0.916 | 1.298 | 1.799 | 28.3 |
| 137 | 1.165 | 0.911 | 1.285 | 1.865 | 30.5 | 28.3 | 1.165 | 0.911 | 1.285 | 1.865 | 28.3 |
| 138 | 1.18 | 0.931 | 1.267 | 1.94 | 30.5 | 28.4 | 1.18 | 0.931 | 1.267 | 1.94 | 28.4 |
| 139 | 1.184 | 0.959 | 1.238 | 1.884 | 30.5 | 28.4 | 1.184 | 0.959 | 1.238 | 1.884 | 28.4 |
| 140 | 1.188 | 0.965 | 1.223 | 1.827 | 30.5 | 28.4 | 1.188 | 0.965 | 1.223 | 1.827 | 28.4 |
| 141 | 1.158 | 0.938 | 1.204 | 1.735 | 30.5 | 28.4 | 1.158 | 0.938 | 1.204 | 1.735 | 28.4 |
| 142 | 1.141 | 0.92 | 1.227 | 1.72 | 30.5 | 28.4 | 1.141 | 0.92 | 1.227 | 1.72 | 28.4 |
| 143 | 1.103 | 0.896 | 1.258 | 1.787 | 30.5 | 28.4 | 1.103 | 0.896 | 1.258 | 1.787 | 28.4 |
| 144 | 1.107 | 0.926 | 1.274 | 1.923 | 30.5 | 28.4 | 1.107 | 0.926 | 1.274 | 1.923 | 28.4 |
| 145 | 1.145 | 0.964 | 1.3 | 1.904 | 30.5 | 28.5 | 1.145 | 0.964 | 1.3 | 1.904 | 28.5 |
| 146 | 1.194 | 0.975 | 1.306 | 1.892 | 30.5 | 28.5 | 1.194 | 0.975 | 1.306 | 1.892 | 28.5 |
| 147 | 1.204 | 0.949 | 1.282 | 1.767 | 30.5 | 28.5 | 1.204 | 0.949 | 1.282 | 1.767 | 28.5 |
| 148 | 1.203 | 0.922 | 1.245 | 1.717 | 30.5 | 28.5 | 1.203 | 0.922 | 1.245 | 1.717 | 28.5 |
| 149 | 1.159 | 0.901 | 1.209 | 1.765 | 30.5 | 28.5 | 1.159 | 0.901 | 1.209 | 1.765 | 28.5 |
| 150 | 1.15 | 0.929 | 1.236 | 1.819 | 30.5 | 28.5 | 1.15 | 0.929 | 1.236 | 1.819 | 28.5 |
| 151 | 1.149 | 0.949 | 1.269 | 1.897 | 30.5 | 28.4 | 1.149 | 0.949 | 1.269 | 1.897 | 28.4 |
| 152 | 1.158 | 0.97 | 1.3 | 1.891 | 30.5 | 28.4 | 1.158 | 0.97 | 1.3 | 1.891 | 28.4 |
| 153 | 1.187 | 0.96 | 1.307 | 1.88 | 30.5 | 28.4 | 1.187 | 0.96 | 1.307 | 1.88 | 28.4 |
| 154 | 1.218 | 0.917 | 1.31 | 1.783 | 30.5 | 28.4 | 1.218 | 0.917 | 1.31 | 1.783 | 28.4 |
| 155 | 1.209 | 0.897 | 1.276 | 1.722 | 30.5 | 28.4 | 1.209 | 0.897 | 1.276 | 1.722 | 28.4 |
| 156 | 1.227 | 0.917 | 1.226 | 1.762 | 30.5 | 28.4 | 1.227 | 0.917 | 1.226 | 1.762 | 28.4 |
| 157 | 1.208 | 0.94 | 1.218 | 1.879 | 30.5 | 28.4 | 1.208 | 0.94 | 1.218 | 1.879 | 28.4 |
| 158 | 1.184 | 0.96 | 1.24 | 1.908 | 30.5 | 28.4 | 1.184 | 0.96 | 1.24 | 1.908 | 28.4 |
| 159 | 1.152 | 0.981 | 1.269 | 1.862 | 30.5 | 28.4 | 1.152 | 0.981 | 1.269 | 1.862 | 28.4 |
| 160 | 1.139 | 0.967 | 1.311 | 1.803 | 30.5 | 28.4 | 1.139 | 0.967 | 1.311 | 1.803 | 28.4 |
| 161 | 1.168 | 0.924 | 1.336 | 1.745 | 30.5 | 28.5 | 1.168 | 0.924 | 1.336 | 1.745 | 28.5 |
| 162 | 1.186 | 0.905 | 1.323 | 1.734 | 30.4 | 28.6 | 1.186 | 0.905 | 1.323 | 1.734 | 28.6 |
| 163 | 1.197 | 0.913 | 1.283 | 1.834 | 30.4 | 28.6 | 1.197 | 0.913 | 1.283 | 1.834 | 28.6 |
| 164 | 1.217 | 0.943 | 1.267 | 1.891 | 30.4 | 28.6 | 1.217 | 0.943 | 1.267 | 1.891 | 28.6 |
| 165 | 1.207 | 0.953 | 1.22 | 1.88 | 30.4 | 28.6 | 1.207 | 0.953 | 1.22 | 1.88 | 28.6 |
| 166 | 1.202 | 0.978 | 1.197 | 1.842 | 30.4 | 28.7 | 1.202 | 0.978 | 1.197 | 1.842 | 28.7 |
| 167 | 1.16 | 0.953 | 1.236 | 1.76 | 30.4 | 28.6 | 1.16 | 0.953 | 1.236 | 1.76 | 28.6 |
| 168 | 1.113 | 0.903 | 1.287 | 1.756 | 30.4 | 28.6 | 1.113 | 0.903 | 1.287 | 1.756 | 28.6 |
| 169 | 1.125 | 0.917 | 1.325 | 1.786 | 30.4 | 28.7 | 1.125 | 0.917 | 1.325 | 1.786 | 28.7 |
| 170 | 1.147 | 0.929 | 1.297 | 1.851 | 30.4 | 28.7 | 1.147 | 0.929 | 1.297 | 1.851 | 28.7 |
| 171 | 1.189 | 0.955 | 1.265 | 1.913 | 30.3 | 28.7 | 1.189 | 0.955 | 1.265 | 1.913 | 28.7 |
| 172 | 1.208 | 0.987 | 1.241 | 1.895 | 30.3 | 28.7 | 1.208 | 0.987 | 1.241 | 1.895 | 28.7 |
| 173 | 1.193 | 0.964 | 1.221 | 1.894 | 30.3 | 28.7 | 1.193 | 0.964 | 1.221 | 1.894 | 28.7 |
| 174 | 1.15 | 0.932 | 1.255 | 1.788 | 30.3 | 28.7 | 1.15 | 0.932 | 1.255 | 1.788 | 28.7 |
| 175 | 1.125 | 0.895 | 1.275 | 1.751 | 30.3 | 28.7 | 1.125 | 0.895 | 1.275 | 1.751 | 28.7 |
| 176 | 1.128 | 0.926 | 1.31 | 1.774 | 30.3 | 28.7 | 1.128 | 0.926 | 1.31 | 1.774 | 28.7 |
| 177 | 1.175 | 0.936 | 1.295 | 1.8 | 30.2 | 28.7 | 1.175 | 0.936 | 1.295 | 1.8 | 28.7 |
| 178 | 1.177 | 0.956 | 1.314 | 1.883 | 30.2 | 28.7 | 1.177 | 0.956 | 1.314 | 1.883 | 28.7 |
| 179 | 1.159 | 0.977 | 1.26 | 1.899 | 30.2 | 28.7 | 1.159 | 0.977 | 1.26 | 1.899 | 28.7 |
| 180 | 1.119 | 0.979 | 1.233 | 1.862 | 30.2 | 28.7 | 1.119 | 0.979 | 1.233 | 1.862 | 28.7 |
| 181 | 1.136 | 0.938 | 1.233 | 1.789 | 30.2 | 28.6 | 1.136 | 0.938 | 1.233 | 1.789 | 28.6 |
| 182 | 1.176 | 0.917 | 1.277 | 1.737 | 30.2 | 28.6 | 1.176 | 0.917 | 1.277 | 1.737 | 28.6 |
| 183 | 1.199 | 0.906 | 1.324 | 1.762 | 30.2 | 28.6 | 1.199 | 0.906 | 1.324 | 1.762 | 28.6 |
| 184 | 1.185 | 0.901 | 1.314 | 1.844 | 30.1 | 28.6 | 1.185 | 0.901 | 1.314 | 1.844 | 28.6 |
| 185 | 1.159 | 0.96 | 1.305 | 1.901 | 30.1 | 28.6 | 1.159 | 0.96 | 1.305 | 1.901 | 28.6 |
| 186 | 1.122 | 0.976 | 1.262 | 1.93 | 30.1 | 28.6 | 1.122 | 0.976 | 1.262 | 1.93 | 28.6 |
| 187 | 1.116 | 0.967 | 1.207 | 1.875 | 30.1 | 28.6 | 1.116 | 0.967 | 1.207 | 1.875 | 28.6 |
| 188 | 1.142 | 0.946 | 1.216 | 1.781 | 30.1 | 28.6 | 1.142 | 0.946 | 1.216 | 1.781 | 28.6 |
| 189 | 1.164 | 0.907 | 1.248 | 1.745 | 30.1 | 28.5 | 1.164 | 0.907 | 1.248 | 1.745 | 28.5 |
| 190 | 1.177 | 0.897 | 1.296 | 1.78 | 30.1 | 28.6 | 1.177 | 0.897 | 1.296 | 1.78 | 28.6 |
| 191 | 1.192 | 0.923 | 1.298 | 1.841 | 30.1 | 28.6 | 1.192 | 0.923 | 1.298 | 1.841 | 28.6 |
| 192 | 1.156 | 0.95 | 1.306 | 1.879 | 30 | 28.6 | 1.156 | 0.95 | 1.306 | 1.879 | 28.6 |
| 193 | 1.152 | 0.972 | 1.278 | 1.879 | 30 | 28.7 | 1.152 | 0.972 | 1.278 | 1.879 | 28.7 |
| 194 | 1.098 | 0.965 | 1.227 | 1.837 | 30 | 28.7 | 1.098 | 0.965 | 1.227 | 1.837 | 28.7 |
| 195 | 1.131 | 0.948 | 1.24 | 1.733 | 30 | 28.8 | 1.131 | 0.948 | 1.24 | 1.733 | 28.8 |
| 196 | 1.167 | 0.941 | 1.282 | 1.743 | 30 | 28.8 | 1.167 | 0.941 | 1.282 | 1.743 | 28.8 |
| 197 | 1.185 | 0.899 | 1.323 | 1.791 | 30 | 28.8 | 1.185 | 0.899 | 1.323 | 1.791 | 28.8 |
| 198 | 1.201 | 0.914 | 1.338 | 1.898 | 30 | 28.9 | 1.201 | 0.914 | 1.338 | 1.898 | 28.9 |
| 199 | 1.153 | 0.976 | 1.272 | 1.917 | 30 | 28.9 | 1.153 | 0.976 | 1.272 | 1.917 | 28.9 |
| 200 | 1.115 | 0.973 | 1.244 | 1.909 | 30 | 28.9 | 1.115 | 0.973 | 1.244 | 1.909 | 28.9 |

Natal mahogany

Forest mahogany

Stamvrug

Figure S1: Mixolab profiles of composite doughs prepared with selected indigenous seed flours

Figure S2: Structural instability observed in bread containing 20% marula flour, showing excessive rise followed by collapse at the sides

**10%**

**15%**

**20%**
